# The efficacy and safety of oral microecological agents as add‐on therapy for atopic dermatitis: A systematic review and meta‐analysis of randomized clinical trials

**DOI:** 10.1002/clt2.12318

**Published:** 2023-12-04

**Authors:** Peiwen Xue, Haiyan Qin, Di Qin, Huilin Liu, Juan Li, Rongjiang Jin, Xianjun Xiao

**Affiliations:** ^1^ School of Health Preservation and Rehabilitation Chengdu University of Traditional Chinese Medicine Chengdu Sichuan China; ^2^ Acupuncture and Tuina School Chengdu University of Traditional Chinese Medicine Chengdu Sichuan China; ^3^ Affiliated Sichuan Provincial Rehabilitation Hospital of Chengdu University of TCM Chengdu Sichuan China

**Keywords:** atopic dermatitis, efficacy, meta‐analysis, microecological agents, safety, systematic review

## Abstract

**Background:**

Atopic dermatitis (AD) is a common skin disease that is hard to completely cure in a short time. Guidelines recommend the use of topical corticosteroids (TCS) as first‐line anti‐inflammatory therapy for AD, but long‐term use has significant side effects. Microecological agents (MA), including probiotics, prebiotics and synbiotics, have been widely reported as a potential adjunctive therapy of AD, but whether MA can contribute to AD treatment is currently controversial. Therefore, we conducted a systematic review and meta‐analysis to investigate whether MA as an add‐on therapy for AD has synergistic and attenuated effects and to further understand the role of MA in clinical interventions for AD.

**Methods:**

We systematically searched Medline, Embase, Web of Science, Cochrane Library and PsycINFO databases up to Apr 11, 2023, and bibliographies were also manually searched, for potentially relevant studies regarding MA as additional therapy of AD. The Cochrane Risk of Bias Tool for assessing risk of bias was used to assess the quality of randomized controlled trials (RCTs). Two reviewers screened studies, extracted data, and evaluated the risk of bias independently. The primary outcomes (SCORAD scores and the number of adverse events) and the secondary outcomes (pruritus scores, the quality of life and the frequency of TCS) were extracted from each article. The data were combined and analyzed to quantify the safety and efficacy of the treatment. R (V4.4.3) software was used for data synthesis. The certainty of the evidence was evaluated with the Grade of Recommendation, Assessment, Development and Evaluation (GRADE) system. We also performed a trial sequential analysis to assess the reliability of the evidence.

**Results:**

A total of 21 studies, including 1230 individuals, were identified, 20 of which met the eligibility criteria for the meta‐analysis. Our pooled meta‐analyses showed that compared with controls, oral MA as an add‐on therapy was associated with significantly lower SCORAD scores (MD = −5.30, 95% CI −8.50, −1.55, *p* < 0.01, *I* ^*2*^ = 81%). However, adverse events, pruritus scores, quality of life, and frequency of TCS use showed no significant difference in this meta‐analysis study (*p* > 0.05).

**Conclusions:**

This meta‐analysis showed that MA plus TCS could be an effective and safe treatment for patients with AD to relieve relevant symptoms, which might be used as an add‐on therapy in the treatment of AD. However, due to the limited number of studies, results should be interpreted with caution. Further studies with a larger sample size are needed to explore the optimal protocol of MA plus TCS.

## 1 INTRODUCTION

Atopic dermatitis (AD), characterized by intense itching, dry skin and redness, is a chronic relapsing inflammatory skin disease. It is one of the most common skin diseases in dermatological practice.^1^ The global prevalence of AD ranged from 15% to 20% in children and up to 10% in adults.^2^ AD had the highest disability adjusted life year (DALY) burden of all skin diseases and ranked the 15th among all nonfatal diseases globally.^3^ In addition, AD is commonly associated with sleep disturbances, negative emotions, and decreased productivity, which seriously affects the patients' quality of life.^4^ AD has become one of the most intractable public health issues worldwide.

The basic management of AD involves emollient therapy, topical therapy, avoiding specific and non‐specific triggers.^5^ The ETFAD/EADV Eczema task force^6^ and the European guidelines^5^ recommended topical corticosteroids (TCS) as the first‐line anti‐inflammatory treatment for AD. Although TCS has been the mainstay of AD treatment, while its long‐term and frequent use is accompanied by several adverse effects, for example, skin atrophy, stria, purpura, hypothalamic pituitary axis suppression and growth suppression.^7^ Inappropriate TCS use may induce topical steroid withdrawal syndrome.^8^ Furthermore, corticophobia was also common in patients with AD who mistakenly believed that the side effects of TCS outweighed its therapeutic benefits.^9^ This would decrease treatment adherence and limit the possibilities for better control of the symptoms. To avoid steroid‐related side‐effects and reduce the lengthy use, application of TCS in combination with other treatment modalities was recommended^6^ by the guidelines.

Microecological agents (MA) including probiotics, prebiotics and synbiotics are able to modulate gut microbiota, improve gut barrier function and relieve symptoms of skin diseases.^10^ Results of clinical studies^11^, ^12^ showed that MA supplementation could improve symptomatology, clinical severity of AD and quality of life. Moreover, researchers suggested that MA had a steroid sparing effect, could be used as an add‐on therapy to TCS.^13^, ^14^ Although studies have been conducted on the effect of MA as an add‐on therapy for AD, these results are conflicting.^12^, ^15^ Besides, MA related adverse events included abdominal pain, diarrhea, etc.^16^ Therefore, we raised the following questions as an add‐on therapy: (1) whether MA plus TCS is more effective than TCS alone? (2) whether MA is able to reduce the accompanying side effects of TCS or the dependence on TCS? (3) what are the influencing factors on the effect of MA?

## 2 MATERIALS AND METHODS

We conducted this systematic review and meta‐analysis according to the A Measurement Tool to Assess Systematic Reviews 2 (AMSTAR 2.0)^17^ and reported conforming to the Preferred Reporting Items for Systematic Evaluations and Meta‐Analyses (PRISMA) guidelines.^18^ The protocol for this systematic review and meta‐analysis was registered in the International Prospective Register of Systematic Reviews (PROSPERO) as CRD42023426811(https://www.crd.york.ac.uk/prospero/#myprospero).

### 2.1 Data sources

We systematically retrieved the randomized controlled trials (RCTs) of MA as an add‐on therapy to TCS for AD from Medline, Embase, Web of Science, Cochrane library, and PsycINFO from their inception to Apr 11, 2023, using the following terms: “Atopic Dermatitis”, “probiotics”, “prebiotics” and “synbiotics”. Furthermore, gray literature, the references of identiﬁed RCTs, relevant reviews and clinical registration websites (ClinicalTrials.gov) were also searched to identify additional RCTs. We consulted experts for possible eligible studies. The entire search strategies were constructed by an experience reviewer (JL) and the detailed search strategies are provided in Appendix 1.

### 2.2 Eligibility criteria

To be included for systematic review: (1) Participants were diagnosed as AD based on the Hanifin and Rajka criteria,^19^ with no limitation of age or gender. (2) Oral probiotics or prebiotics or synbiotics were used as an add‐on therapy to TCS. (3) The comparison included TCS alone, or TCS in combination with placebo. (4) The primary outcomes were SCORAD scores and adverse events. Secondary outcomes were pruritus scores, the frequency of TCS and quality of life. Among which, SCORAD scores or adverse events were obligatory. (5) Double‐blind RCTs. (6) Full texts were available. (7) Studies were published in English.

To be excluded for systematic review: (1) data were inaccessible, (2) non‐double‐blinded cross‐over RCTs, reviews, duplicated publications, commentaries, case reports, case series, observational studies, comments, or in vitro studies.

### 2.3 Studies selection

Endnote X9 was used to manage the retrieved records. After removing duplicates, two independent reviewers (HYQ and PWX) screened titles and abstracts based on inclusion and exclusion criteria. Then, full texts were reviewed to determine eligible studies. After that, the included studies were cross‐examined. Any disagreement was resolved by consulting a third reviewer (JL).

### 2.4 Data collection and extraction

Two reviewers (DQ and PWX) independently extracted data using a predefined data extraction form. The following data were extracted: (1) study information: first author, publication year, and country; (2) participant characteristics: severity, sample size, and age; (3) details of interventions and comparators: types, regimens (dose and dosage form) and duration; (4) primary outcomes and secondary outcomes; and (6) Main results. Any discrepancies were arbitrated by a third reviewer (JL).

With regards to missing data, the corresponding authors were contacted via email. If the data were not displayed by mean and standard deviation, the formula recommended by the Cochrane handbook^20^, ^21^ was used to convert the data. In the case of the data presented in graphs, the semi‐automated extraction tool WebPlotDigitizer

(https://automeris.io/WebPlotDigitizer/, Version 4.3) was utilized to extract data.^22^, ^23^

### 2.5 Risk of bias assessment

The revised Cochrane risk‐of‐bias tool for randomized trials (ROB 2.0) was used to evaluate the risk of bias from five domains: randomization process, deviation from the intended intervention, missing outcome data, measurement of the outcome, and selection of the reported results.^24^ Each domain was judged as “low risk,” “some concerns,” or “high risk”. Two reviewers (PWX and HLL) independently assessed the risk of bias and then cross‐checked. After cross‐examination, disagreements were settled through consultation with an experienced reviewer (RJJ).

### 2.6 Statistical analysis

Among the included studies, different measurement tools were used to evaluate the pruritus scores and quality of life; thus, the standardized mean difference (SMD) was calculated. The SCORAD scores and frequency of TCS were evaluated using the same tools, therefore mean difference (MD) was used. The risk difference (RD) was calculated due to no adverse events. The uncertainty was expressed with 95% confidence intervals (CIs).

Heterogeneity was measured using the chi‐squared test and *I* ^*2*^ statistic.^25^ *p* < 0.05 or *I* ^*2*^ values > 50% was considered significant heterogeneity. A random effects model was used to obtain a more conservative estimate. Forest plots were utilized to present the pooled results. R software (version 4.4.2) was used for data synthesis.

### 2.7 Subgroup analysis

Subgroup analyses were conducted according to the types of MA^26^ (a single strain of probiotic, the mixture of probiotic strains, prebiotics or synbiotics), the age of participants^27^ (infants, children or adults) and the severity of AD^28^ (mild to moderate, moderate or moderate to severe).

### 2.8 Meta regression analysis

Univariable meta‐regression analyses were used to investigate potential sources of heterogeneity based on the characteristics of the study (MA types, participants' age, and AD severity).

### 2.9 Sensitivity analysis

We performed sensitivity analyses by eliminating studies one by one to verify the robustness of the pooled results.

### 2.10 Publication bias

We used a funnel plot and Egger's test to detect publication bias when ≥10 studies with the same outcome were included in the analysis.

### 2.11 TSA

We assessed the risk of false positives or false negatives for primary outcomes by TSA (version 0.9.5.10‐Beta) in a meta‐analysis.^29^ Random effects model with a maximum type I error of 5%, and a maximum type II error of 20% (80% power) were applied. When the cumulative Z‐curve enters the useless zone or crosses the trial sequential monitoring boundary, the expected intervention effect may reach an adequate level of evidence. If the Z‐curve does not cross any boundaries and does not reach the required size of information, it indicates that the evidence is insufficient to draw a conclusion.

### 2.12 Certainty of evidence

We applied the Grading of Recommendations Assessment, Development, and Evaluation (GRADE)^30^ tool to assess the certainty of evidence. Each outcome was evaluated based on the following five aspects: limitations, inconsistency, indirectness, imprecision, and publication bias. The certainty of evidence was accordingly graded as “high,” “moderate,” “low,” or “very low”.

## 3 RESULTS

### 3.1 Characteristics of included studies

**FIGURE 1:**
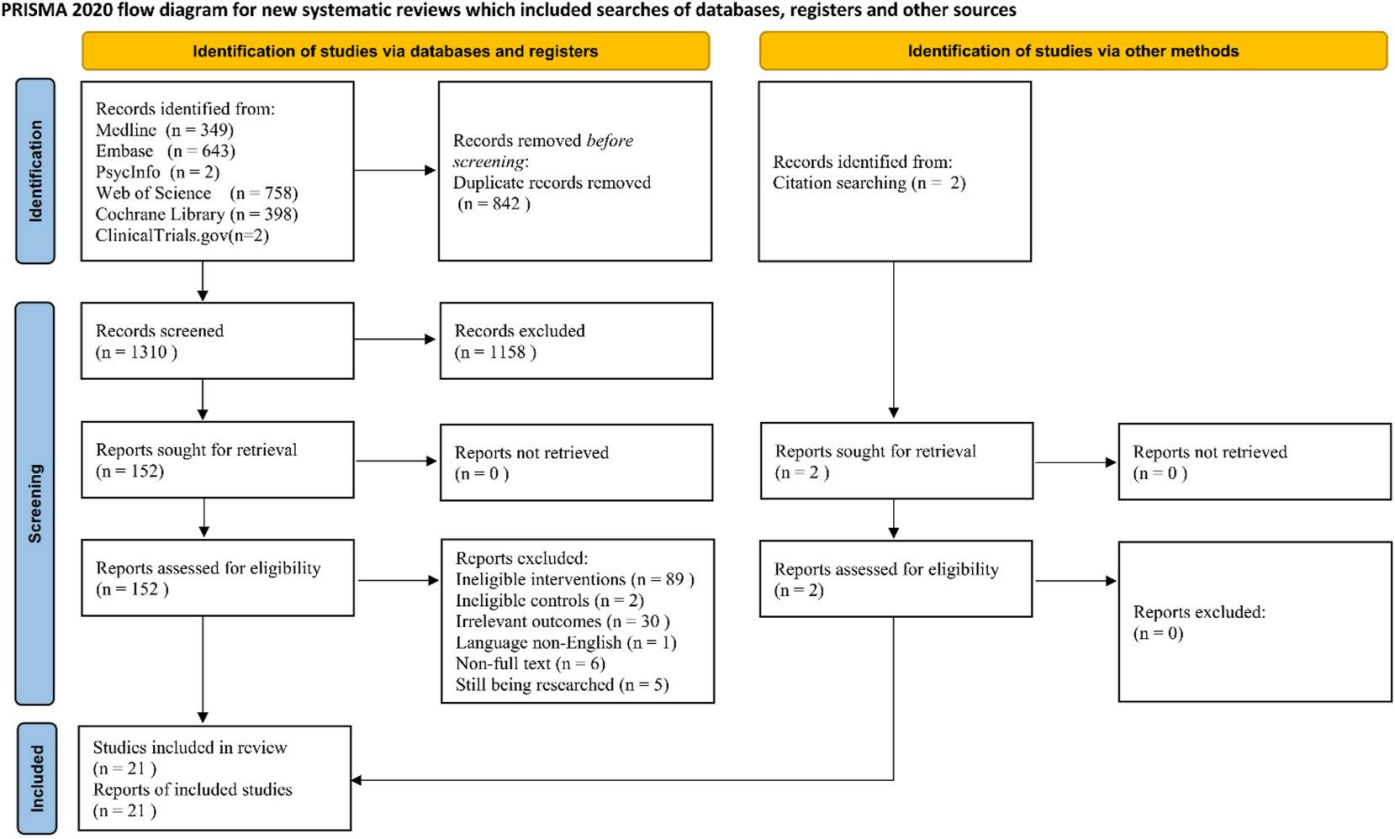
Flow chart for selection of eligible studies.

As shown in Figure 1, a total of 2152 potentially eligible articles were identified. The list of excluded literature and the reasons for exclusion are shown in Appendix 2. Twenty‐one studies involving 1230 patients with AD (622 subjects in the intervention group and 608 subjects in the control group) were finally included in the meta‐analysis.^12^, ^13^, ^14^, ^16^, ^31^, ^32^, ^33^, ^34^, ^35^, ^36^, ^37^, ^38^, ^39^, ^40^, ^41^, ^42^, ^43^, ^44^, ^45^, ^46^, ^47^ The included RCTs were conducted in 12 countries (Australia, Brazil, China, Danish, Germany, India, Iran, Italy, Japan, Korea, Spain and Ukraine).

Fourteen studies adopted a single strain of probiotics. Among them, 13 used *Lactobacillus* (*L*) species (L rhamnosus, L paracasei, L acidophilus, L plantarum, L fermentum, L sakei)^13^, ^16^, ^32^, ^34^, ^35^, ^36^, ^39^, ^40^, ^43^, ^44^, ^45^, ^46^, ^47^ and one used Bifidobacterium species (B lactis).^33^ Four used a mixture of probiotic strains, which were *Lactobacillus* species combined with Bifidobacterium species.^12^, ^14^, ^41^, ^42^ Two studies used synbiotics (probiotic plus prebiotic).^31^, ^37^ One study used prebiotics (kestose).^38^

The reported outcomes involved SCORAD scores,^12^, ^13^, ^14^, ^16^, ^35^, ^37^, ^38^, ^39^, ^42^, ^43^, ^44^, ^45^, ^47^ adverse events,^12^, ^16^, ^31^, ^32^, ^33^, ^34^, ^39^, ^40^, ^42^, ^43^, ^46^, ^47^ pruritus scores,^16^, ^33^, ^34^ the quality of life^33^, ^34^, ^42^, ^47^ and the frequency of TCS.^42^, ^43^ The detailed characteristics of the included studies are shown in Table 1.

**TABLE 1:** Characteristics of the included studies.

Study, country	Number (intervention/control)	Age	Severity	Types of probiotics	Intervention	Comparison	Treatment regimen	Outcomes	Main results
Aldaghi et al. 2022 (Iran)	27/27	＜1 year	NA	Synbiotic (*Lactobacillus* rhamnosus, *Lactobacillus* reuteri, Bifidobacterium infantis, Fructooligosaccharides)	Synbiotic + routine treatment	Routine treatment (topical corticosteroids, emollient (eucerin) and antihistamines)	Liquid	Adverse events	Children and adolescents with AD presented a significant clinical response after 6 months with a mixture of probiotics (*Lactobacillus* rhamnosus, *Lactobacillus* acidophilus, *Lactobacillus* paracasei, and Bifidobacterium lactis. However, this clinical benefit is related to treatment duration. Probiotics should be considered as an adjuvant treatment for AD.
							5 drops/day		
							8 weeks		
D'Auria et al. 2021 (Italy)	26/27	6–36 months	Moderate to severe	*Lactobacillus* paracasei CBA L74	*Lactobacillus* paracasei CBA L74 + topical Corticosteroid, moisturizers	Placebo + topical Corticosteroid, moisturizers	Beverage or liquid food	SCORAD scores	The present study did not prove the efficacy of a fermented rice flour obtained from heat treated *Lactobacillus* paracasei CBA L74 as a complementary approach in significantly reducing AD severity. However, heated killed *Lactobacillus* paracasei CBA L74 showed a corticosteroid sparing effect beyond the treatment period. This issue deserves further and more specific investigations in the light of the growing interest for steroid‐sparing strategies.
							8 g	Sparing effect	
							12 weeks		
Feito‐Rodriguez et al. 2023 (Spain)	35/35	4–17 years	Moderate	Mixture of probiotic strains (Bifidobacterium lactis, Bifidobacterium longum and *Lactobacillus* casei)	Mixture of probiotic strains + topical corticosteroids methylprednisolone aceponate or diflucortolone valerate	Mixture of probiotic strains + topical corticosteroid	Capsules	SCORAD scores	The probiotic used in this clinical trial demonstrates efficacy on the change of the activity index of AD compared to placebo. The total number of days and total amount of topical corticosteroids required by the subjects in the probiotic group showed a significant reduction compared to placebo between 6 and 12 weeks.
							1*10^9 cfu		
							12 weeks		
Folster‐Holst et al. 2006 (Germany)	26/27	1–55 months	Moderate to severe	L. Rhamnosus strain GG	LGG + topical corticosteroid and antihistamines	Placebo + topical corticosteroid and antihistamines	Capsules	SCORAD scores	The results could not confirm LGG as an effective treatment of AD in infancy. However, there might still be subgroups of patients suitable for a probiotic intervention. In particular, the role of IgE‐mediated sensitization as a prerequisite for treatment success remains to be studied. Due to a broad range of prebiotic and probiotic formulas and/or foods it may be difficult to detect therapeutic effects unless particular care is taken to eliminate bias.
							5*10^9 cfu	Adverse events	
							8 weeks	Pruritus scores	
								Sparing effect	
Inoue et al. 2014 (Japan)	24/25	＞16 years	NA	*Lactobacillus* acidophilus L‐92	*Lactobacillus* acidophilus L‐92 + topical corticosteroid moisturizer and one or two oral antihistamines	Placebo + topical corticosteroid moisturizer and one or two oral antihistamines	tablet	Adverse events	The trial demonstrated that L‐92 is effective against AD in adults. No serious side effects were observed in any of the patients. The study suggested that L‐92 could be used as a food supplement to reduce the dose of steroidal anti‐inflammatory ointments required for atopic treatment. However, further studies with different strains of *Lactobacillus* are necessary to confirm its beneficial role in AD and to clarify the immunological mechanisms.
							20.7 mg/day		
							8 weeks		
Matsumoto et al. 2014 (Japan)	22/22	NA	Moderate to severe	Bifidobacterium animalis subsp lactis LKM512	LKM512 + topical corticosteroid	Placebo + topical corticosteroid	Capsules	Adverse events	The probiotic LKM512 strain seems to be effective at reducing pruritus and improving QOL score in the symptom category in adult patients with AD.
							6*10^9 cfu	Pruritus scores	
							8 weeks	Quality of life	
Moroi et al. 2011 (Japan)	16/17	20–65 years	Mild to moderate	*Lactobacillus* paracasei K71	*Lactobacillus* paracasei K71 + corticosteroid and tacrolimus	Placebo + corticosteroid and tacrolimus	Powder	Adverse events	The findings suggest that either viable or inactivated probiotic lactobacilli may exhibit beneficial activities in the management of symptomatic AD. Further studies on the clinical usefulness of a LAB diet as a complementary modality for AD patients with standard treatments are warranted to be continued in more detail.
							2*10^11 cfu	Pruritus scores	
							12 weeks	Quality of life	
Navarro‐Lopez et al. 2018 (Spain)	23/24	4–17 years	Moderate	Bifidobacterium lactis CECT 8145, B Longum CECT 7347, and *Lactobacillus* casei CECT 9104 and maltodextrin	Mixture of probiotic strains + topical methylprednisolone aceponate, moisturizer, and 1 oral antihistamine	Placebo + topical methylprednisolone aceponate, moisturizer, and 1 oral antihistamine	Tablet	SCORAD scores	The results of our study indicate a strong positive effect in reducing the SCORAD index and use of topical corticosteroids in the group treated with the probiotic mixture. This evidence supports the efficacy of administering this probiotic mixture to patients with moderate AD and suggests that it could be used more extensively in clinical practice.
							1*10^9 cfu	Adverse events	
							12 weeks		
Prakoeswa et al. 2017 (India)	12/10	0–14 years	Mild and moderate	*Lactobacillus* plantarum IS‐10506	*Lactobacillus* plantarum IS‐10506 + standard treatment	Placebo + standard treatment(antihistamines, emollients, and topical corticosteroids)	Capsules	SCORAD scores	Probiotic L. plantarum IS‐10506 showed ability to reduce clinical symptoms in AD children, as shown by a decrease in SCORAD. Probiotics L. plantarum IS‐10506 is a potential treatment for preventing recurrence or progression to chronic AD in children who are unable to eliminate allergenic ingredients and the emphasis on alternative therapies through the induction of immunological tolerance.
							1*10^9 cfu		
							12 weeks		
Rosenfeldt et al. 2003 (Danish)	20/23	1–13 years	NA	*Lactobacillus*	*Lactobacillus* strains + topical corticosteroids	Placebo + topical corticosteroid	Powder	SCORAD scores	A combination of L rhamnosus 19,070‐2 and L reuteri DSM 122460 was beneficial in the management of AD. Administration of probiotic *Lactobacillus* strains (a mixture of L rhamnosus 19,070‐2 and L reuteri DSM 12246) to children with AD was associated with a moderate improvement in the clinical severity of eczema.
							1*10^9 cfu		
							6 weeks		
Shaflei et al. 2011 (Iran)	20/21	1–36 months	Moderate to severe	Synbiotic (probiotic plus prebiotic)	Synbiotic + bathing habits, moisturing cream (Eucerin) and topical corticosteroid	Placebo + bathing habits, moisturing cream (Eucerin) and topical corticosteroid	Powder	SCORAD scores	The results could not confirm synbiotic as an effective treatment for childhood AD. However, probiotics and synbiotics may have a potential role in the treatment of atopic dermatitis, but studies to date have not been persuasive. There may be special subset which would be responsive to probiotic or synbiotic but further studies are needed to determine these suitable subgroups.
							1*10^9 cfu		
							8 weeks		
Shibata et al. 2009 (Japan)	15/15	＜3 years	NA	Kestose	Kestose + topical corticosteroid	Placebo + topical corticosteroid	Oral	SCORAD scores	Kestose was found to exert a beneficial effect on the clinical symptoms in infants with AD. In a comparison between groups at the same timepoint, the SCORAD scores in the kestose group were significantly lower than the scores in the placebo group
							1–2g		
							12 weeks		
Weston et al. 2005 (Australia)	28/28	6–18 months	Moderate to severe	*Lactobacillus* fermentum VRI‐033 PCC	*Lactobacillus* fermentum VRI‐033 PCC + topical corticosteroid	Placebo + topical corticosteroid	Powder	SCORAD scores	Supplementation with probiotic L fermentum VRI‐003 PCC is beneficial in improving the extent and severity of AD in young children with moderate or severe disease.
							10*10^9 cfu	Adverse events	
							8 weeks		
Yamamoto et al. 2016 (Japan)	28/29	＞16 years	Mild to moderate	*Lactobacillus* acidophilus L‐92	*Lactobacillus* acidophilus L‐92 + topical corticosteroid	Placebo + topical corticosteroid	Tablet	Adverse events	In chronic progression of AD, AD symptoms could be improved when long‐term intake of the L‐92 strain was combined with prescribed medications.
							20.7 mg		
							24 weeks		
Andrade et al. 2022 (Brazil)	24/16	6 months to 19 years	NA	A mixture of probiotics (*Lactobacillus* rhamnosus, *Lactobacillus* acidophilus, *Lactobacillus* paracasei, Bifidobacterium lactis)	A mixture of probiotics + standard treatment (topical corticosteroid)	Placebo + standard treatment	Oral	SCORAD scores	The study demonstrated that children and adolescents with AD treated with a combination of probiotics for 6 months presented a statistically significant reduction of SCORAD and used less topical immunosuppressants as compared to the placebo group. This reduction persisted for 3 months after the treatment has been discontinued.
							1*10^9 cfu		
							24 weeks		
Gerasimov et al. 2010 (Ukraine)	43/47	1–3 years	Moderate to severe	A mixture of L. acidophilus DDS‐1, B. lactis UABLA‐12 with fructo‐oligosaccharide	A mixture of probiotics + topical corticosteroid	Placebo + topical corticosteroid	Powder	SCORAD scores	The administration of a probiotic mixture containing L. acidophilus DDS‐1, B. lactis UABLA‐12, and fructo‐oligosaccharide was associated with significant clinical improvement in children with AD. Probiotic showed a greater decrease in the SCORAD, IDQOL and DFI scores than did children from the placebo group. Use of topical corticosteroids during the 8‐week trial period less than probiotic patients. The efficacy of probiotic therapy in adults with AD requires further investigation.
							5*10^9 cfu	Adverse events	
							8 weeks	Quality of life	
								Sparing effect	
Gruber et al. 2007 (Germany)	54/48	3–12 months	Mild to moderate	*Lactobacillus* rhamnosus GG	LGG + topical corticosteroid	Placebo + topical corticosteroid	Capsules	SCORAD scores	This placebo‐controlled trial showed no therapeutic effect of LGG against mild‐to‐moderate atopic dermatitis in infancy.
							5*10^9 cfu	Adverse events	
							12 weeks	Sparing effect	
Han et al. 2012 (Korea)	44/39	12 months to 13 years	Mild to moderate	*Lactobacillus* plantarum CJLP133	*Lactobacillus* plantarum CJLP133 + topical corticosteroid	Placebo + topical corticosteroid	Oral	SCORAD scores	The SCORAD score at week 14 was lower in the probiotic group than in the placebo group. No statistical differences in the total use of topical corticosteroids were found between two groups. Supplementation with probiotic L. plantarum CJLP133 is beneficial in the treatment of AD in children. However, its long‐term effect remains unclear, as L. plantarum does not persist in the gut. The exact mechanism by which the probiotics modulate the immune system also needs to be studied.
							5*10^9 cfu	Sparing effect	
							12 weeks		
Woo et al. 2010 (Korea)	41/34	2–10 years	Moderate to severe	*Lactobacillus* sakei	*Lactobacillus* sakei + topical corticosteroid	Placebo + topical corticosteroid	Oral	SCORAD scores	This study provides evidence that administration of the probiotic strain L sakei may be associated with improvement in the clinical severity of AD. But the effect of probiotic supplementation seemed to be selective because levels of responsiveness were different between patients receiving the probiotic. A prolonged period of observation is needed to provide more convincing evidence of the efficacy of L sakei in AD.
							5*10^9 cfu	Sparing effect	
							12 weeks		
Wu et al.2017 (China)	30/32	4–48 months	Moderate to severe	*Lactobacillus* rhamnosus	*Lactobacillus* rhamnosus + topical corticosteroid	Placebo + topical corticosteroid	Capsules	Adverse events	LR was effective to decrease symptoms of atopic dermatitis. Subjects who took LR for 8 weeks expressed less SCORAD in the three components: Area of affected skin, intensity of atopic dermatitis, and subject symptoms, with a significant decrease in the mean change of intensity from baseline compared with placebo
							350 mg	Sparing effect	
							8 weeks		
Yan et al.2019 (China)	64/62	4–30 months	Moderate to severe	*Lactobacillus* paracasei GM‐080	GM080 + topical corticosteroid	Placebo + topical corticosteroid	Powder	SCORAD scores	The probiotic L. paracasei was not beneficial as a complementary approach to topical corticosteroids in infants with AD. However, slight beneficial effects may have been masked by the moderate potency corticoid.
							10*10^9 cfu	Adverse events	
							16 weeks	Quality of life	
								Sparing effect	

### 3.2 Risk of bias assessment

**FIGURE 2:**
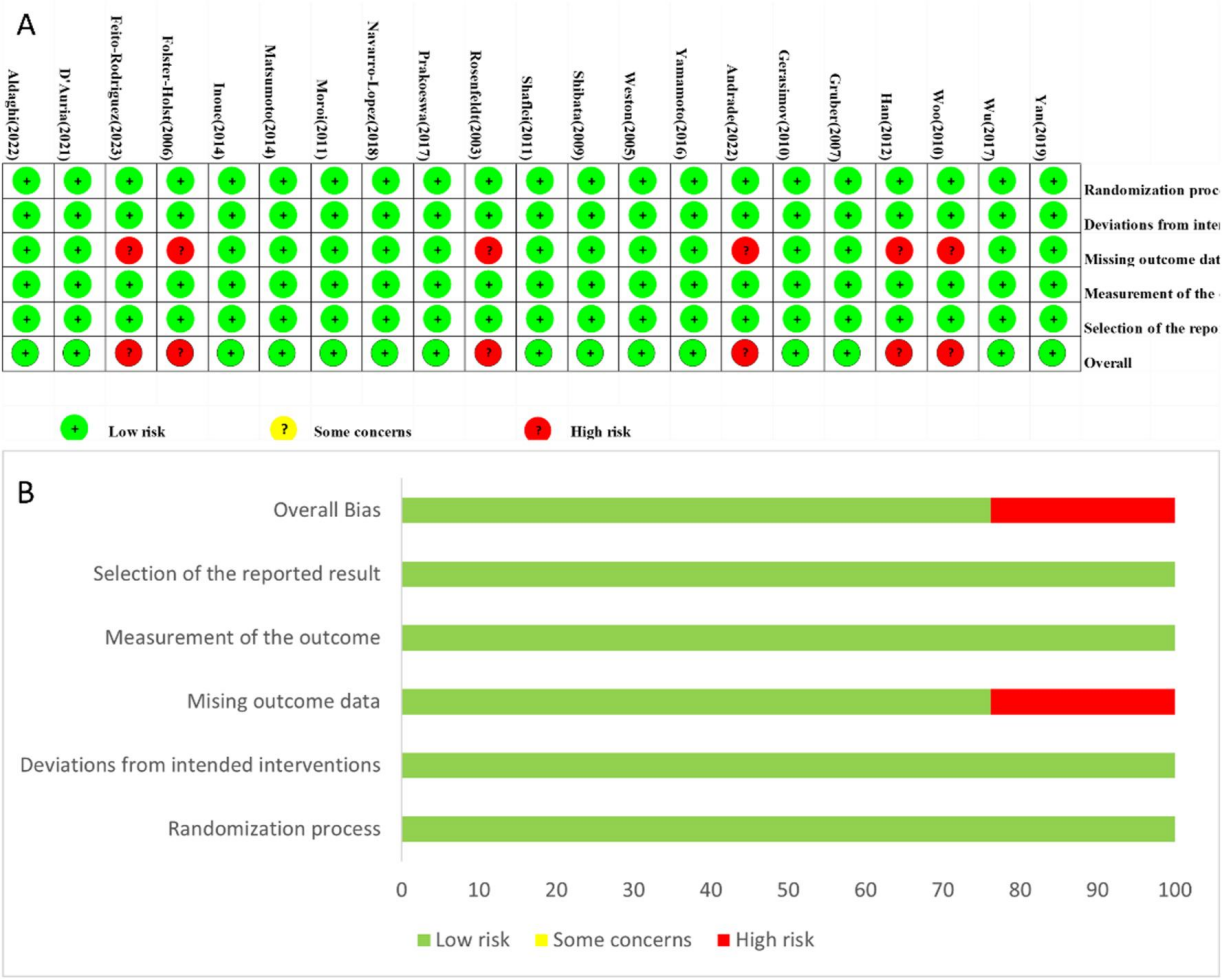
(A) The plot of RoB 2.0 for each included study. (B) Proportions of individual study for each domain.

The plot of the risk of bias (RoB 2.0) for each included study is presented in Figure 2A, and the proportions of individual studies are presented in Figure 2B. In the randomization process, deviation from intended interventions, measurement of outcomes and selection of the reported result, all trials were rated as low risk of bias. For missing outcome, six studies^14^, ^16^, ^36^, ^41^, ^44^, ^45^ with high dropout rates and did not report the details of drop‐outs, which were rated as high risk of bias. In summary, the overall risk of bias in 15 trials was considered low risk and six trials were considered high.

### 3.3 Results of meta‐analysis

#### 3.3.1 SCORAD scores

**FIGURE 3:**
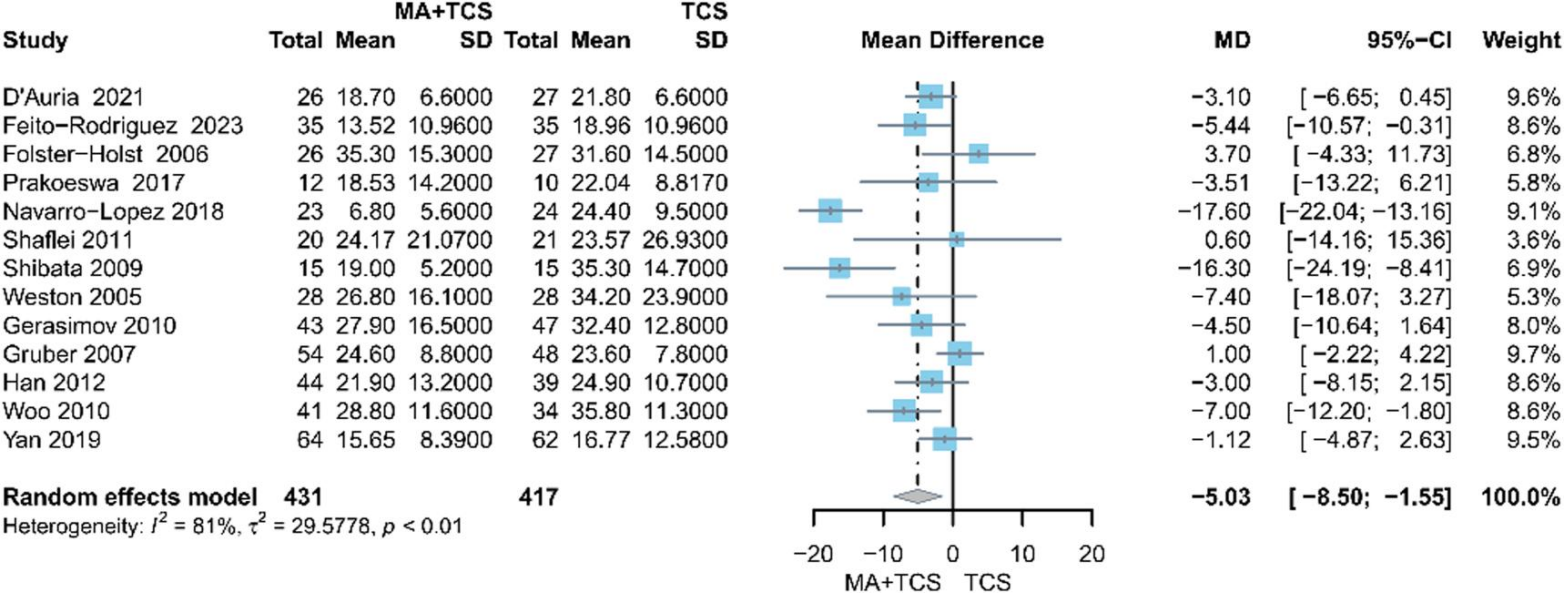
Forest plot of SCORSD scores.

Thirteen studies reported the SCORAD scores^12^, ^13^, ^14^, ^16^, ^35^, ^37^, ^38^, ^39^, ^42^, ^43^, ^44^, ^45^, ^47^; the synthesized result showed that oral MA plus TCS was more effective than TCS in relieving SCORAD scores (MD = −5.30, 95% CI −8.50 to −1.55, *p* < 0.01, *I* ^*2*^ = 81%) (Figure 3).

#### 3.3.2 Adverse events

**FIGURE 4:**
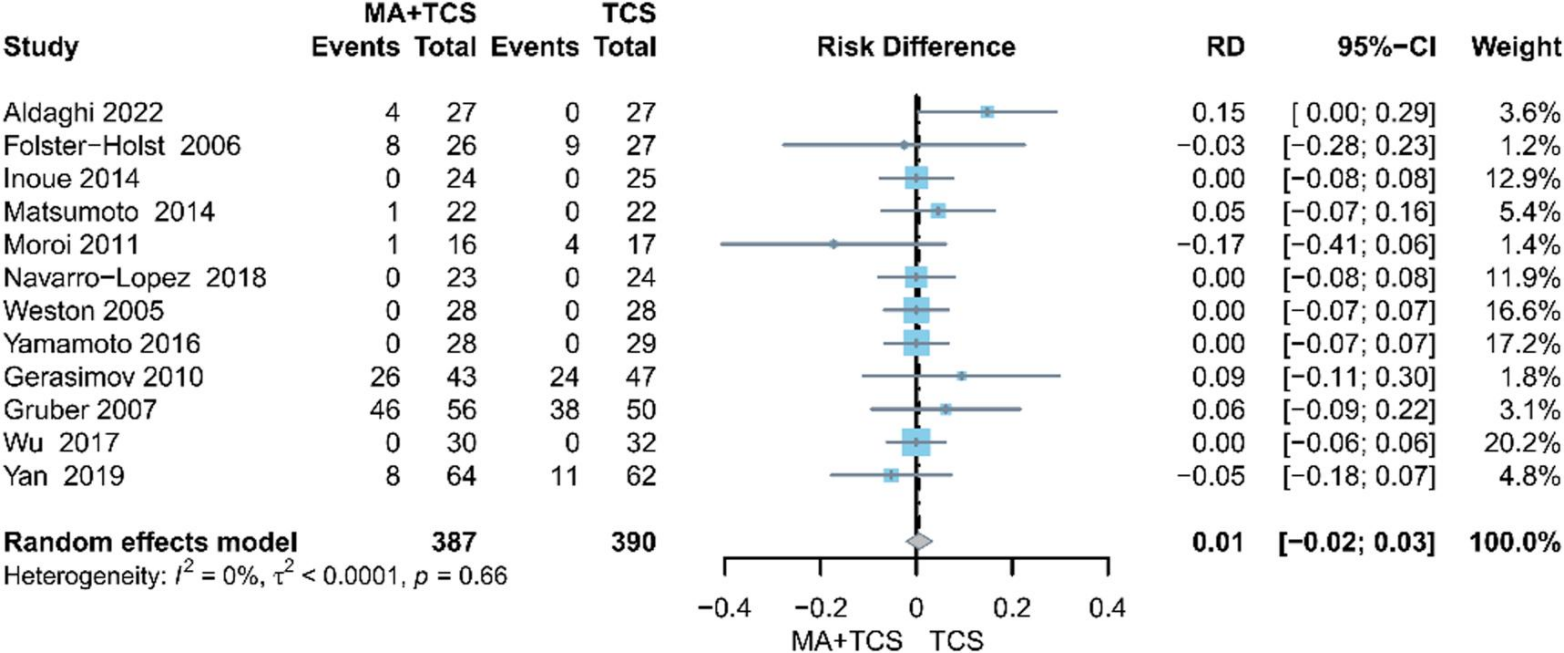
Forest plot of adverse events.

The pooled result of 12 studies on adverse events^12^, ^16^, ^31^, ^32^, ^33^, ^34^, ^39^, ^40^, ^42^, ^43^, ^46^, ^47^ indicated that there was no statistical difference between MA plus TCS and TCS (RD 0.01, 95% CI −0.02 to 0.03, *p* = 0.66, *I* ^*2*^ = 0%) (Figure 4).

#### 3.3.3 Pruritus scores

**FIGURE 5:**
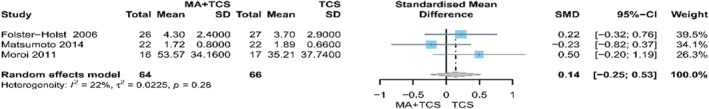
Forest plot of pruritus scores.

The overall result of pruritus scores^16^, ^33^, ^34^ showed no difference between MA plus TCS and TCS (SMD 0.14, 95% CI −0.25 to 0.53, *p* = 0.48, *I* ^*2*^ = 22%) (Figure 5).

#### 3.3.4 Quality of life

**FIGURE 6:**
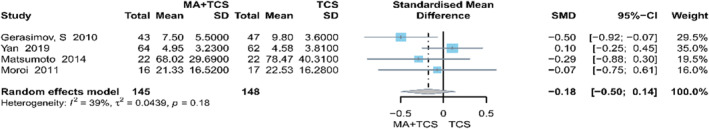
Forest plot of quality of life.

Four studies reported the quality of life.^33^, ^34^, ^42^, ^47^ No difference was detected between MA plus TCS and TCS in quality of life (SMD −0.18, 95% CI −0.50 to 0.14, *p* = 0.26, *I* ^*2*^ = 39%) (Figure 6).

#### 3.3.5 The frequency of TCS

**FIGURE 7:**
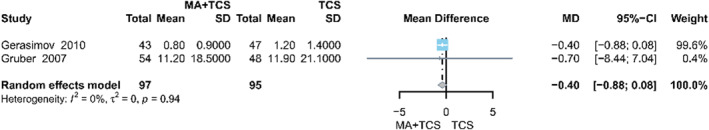
Forest plot of the frequency of TCS.

The result demonstrated that MA plus TCS was not superior to TCS (MD −0.40, 95% CI −0.88 to 0.08, *p* = 0.1, *I* ^*2*^ = 0%) (Figure 7).

### 3.4 Results of subgroup analysis

We conducted subgroup analyses according to different MA types (Figure S1 and S2 in Appendix 3), participants' age (Figure S3 and S4 in Appendix 3) and AD severity (Figure S5 and S6 in Appendix 3).

#### 3.4.1 Subgroup of different MA types

In the outcome of SCORAD scores (Table 2), oral mixtures of probiotic strains and prebiotics were more effective than TCS. Furthermore, oral mixtures of probiotic strains were better than other MA types. In terms of adverse events (Table 3), all types of the MA were not superior to TCS, except synbiotic.

**TABLE 2:** Subgroup analysis of SCORAD.

Factor	Number of studies	Number (intervention/control)	SD (95%CI)	*p* value	*I* ^*2*^
MA types					
*Lactobacillus*	8	295/275	−2.07(−4.31 to 0.16)	0.07	34%
Mixted strains	3	101/106	−9.35(−17.76 to 0.94)	0.04	88%
Prebiotic	1	20/21	0.60(−14.16 to 15.36)	＜0.01	‐
Synbiotic	1	15/15	−16.30(−24.19 to 8.41)	0.94	‐
Subgroup differences				＜0.01	
Participants' age					
Infants	8	276/275	−3.11(−7.06 to 0.84)	0.09	65%
Children	5	155/142	−7.67(−13.24 to −2.10)	0.01	83%
Subgroup differences				0.19	
AD severity					
Mild and moderate	3	110/97	−0.74(−4.08 to 2.59)	0.75	5%
Moderate	3	73/74	−12.09(−20.83 to −5.15)	＜0.01	85%
Moderate to severe	7	248/246	−2.94(−5.15 to −0.74)	0.01	15%
Subgroup differences				0.02	

**TABLE 3:** Subgroup Analysis of Adverse events.

Factor	Number of studies	Number (intervention/control)	RD (95%CI)	*p* value	*I* ^*2*^
MA types					
*Lactobacillus*	8	272/270	−0.00(−0.04 to 0.03)	0.79	0%
Bifidobacterium	1	22/22	0.05(−0.07 to 0.16)	0.45	‐
Mixted strains	2	66/71	0.01(−0.06 to 0.09)	0.69	0%
Synbiotic	1	27/27	0.15(0.00 to 0.29)	0.04	‐
Subgroup differences				0.20	
Participants' age					
Infants	7	274/273	0.01(−0.03 to 0.05)	0.52	0%
Adults	4	90/93	0.00(−0.04 to 0.05)	0.99	0%
Children	1	23/24	0.00(−0.08 to 0.08)	1	‐
Subgroup differences				0.92	
AD severity					
Mild and moderate	3	100/96	−0.00(−0.06 to 0.06)	0.89	26%
Moderate	2	50/51	0.06(−0.08 to 0.20)	0.47	68%
Moderate to severe	6	213/218	0.00(−0.04 to 0.04)	0.89	0%
No information	1	24/25	0.00(−0.08 to 0.08)	1	‐
Subgroup differences				0.88	

#### 3.4.2 Subgroup of different participants' age

Oral MA reduced the SCORAD scores (Table 2) in children but had no effect on infants. With regard to adverse events (Table 3), there were no statistical differences between MA + TCS and TCS in AD patients at different ages.

#### 3.4.3 Subgroup of different AD severity

The results of subgroup analysis demonstrated that oral MA plus TCS had an effect on decreasing SCORAD scores in moderate and moderate to severe AD patients (Table 2). However, there was no effect on adverse events among AD patients with different severity (Table 3).

### 3.5 Sensitivity analysis

**FIGURE 8:**
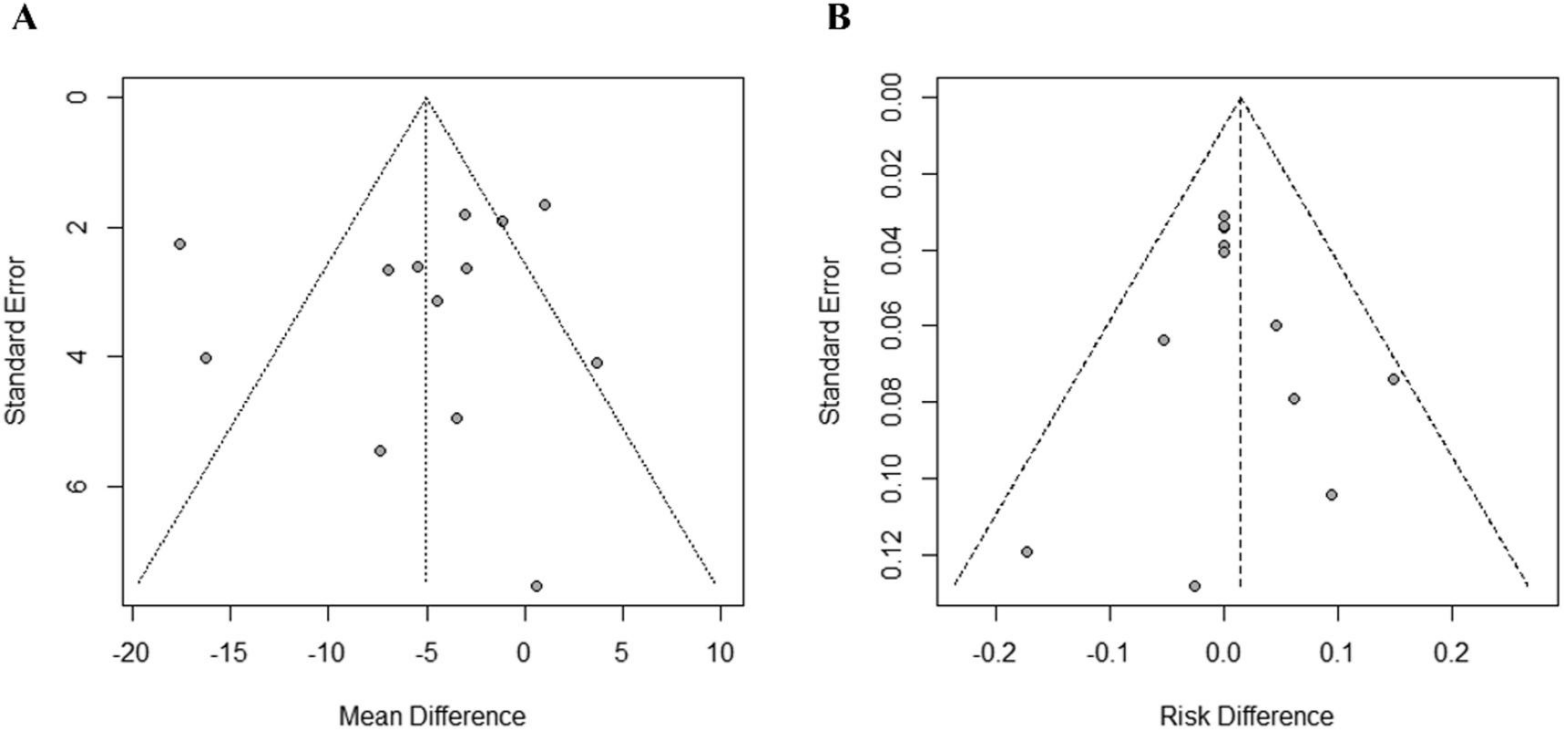
(A) Funnel plot of SCORSD scores. (B) Funnel plot of adverse events.

The results of SCORAD scores and adverse events did not alter after excluding studies one by one (Figures 7‐8 in Appendix 3).

### 3.6 Results of meta regression analysis

The results of univariate meta‐regression showed that the types of MA and the severity of AD were the sources of heterogeneity (Table 4).

**TABLE 4:** Results of univariable meta‐regression.

Factor	Number of studies	Regression coefficient (95% CI)	Standard error<	*p* value
MA types				
*Lactobacillus*	8	1		
Mixted strains	3	−7.3374(−13.5921 to −1.0827)	3.1912	0.0215
Prebiotic	1	−14.0172(−25.4097 to −2.6247)	5.8126	0.0159
Synbiotic	1	2.8828(−14.0126 to 19.7783)	8.6203	0.7381
Participants' age				
Children	5	1		
Infants	8	4.5450(−2.1666 to 11.2566)	3.4244	0.1844
AD severity				
Mild and moderate	3	1		
Moderate	3	−11.6649(−18.6601 to −4.6697)	3.5691	0.0011
Moderate to severe	7	−1.7308(−7.7570 to 4.2954)	3.0747	0.5735

### 3.7 Publication bias

The funnel plot and *Egger's* test of SCORAD scores and adverse events suggested that no evidence of publication bias existed (Figuress 8A–B).

### 3.8 TSA

Although the cumulative sample size did not meet expectations, the Z‐curve of SCORAD crossed both the conventional and TSA boundaries, which indicated that MA, as an add‐on therapy to TCS, was superior to TCS (Figure S9, Appendix 3). The included sample size of adverse events did not achieve the required information size, and its cumulative Z‐curve did not cross either the conventional boundaries or the boundaries of the TSA. Thus, more studies are needed to verify the safety of MA as an addition therapy (Figure S10, Appendix 3).

### 3.9 Certainty of evidence

The results of certainty of evidence are shown in Figure S11, Appendix 3. The adverse events were rated as high certainty of evidence. The certainty of evidence for pruritus scores, quality of life and the frequency of TCS were graded as moderate, while the evidence of the SCORAD was rated as low certainty. The reasons for downgrading were mainly attributed to the risk of bias of the included studies, inconsistency and imprecision.

## 4 DISCUSSION

In the present study, the results showed that MA as an add‐on therapy was better than TCS in lowering the SCORAD scores. However, the high heterogeneity (*I* ^*2*^ = 81%) was detected, which should be treated with caution. The results of meta‐regression indicated that different MA types and AD severity were the reasons for heterogeneity. The minimum clinically important difference (MCID) is the smallest change in the outcome measure and refers to a clinically relevant outcome.^48^ Researchers^49^, ^50^ reported that the reduction of the SCORAD index to more than 8 units was considered as MCID. The pooled result of SCORAD scores in this study was a reduction of 5 units. It might be associated with the inconsistency of disease severity and probiotic strains.^51^ According to the results of subgroup analysis on MA types, oral mixed strains and synbiotic decreased the SCORAD index by 9.35 and 16.3 units, respectively. With regard to AD severity, the SCORAD index in patients with moderate AD decreased 12.09 units. The results of adverse events indicated that MA plus TCS was a safe way to treat AD. There were no differences in pruritus, quality of life, and frequency of TCS using in comparison of MA plus TCS versus TCS.

It was reported that immune regulation and anti‐inflammatory effects of MA played an important role in the treatment of AD.^52^ Rosenfeldt et al^36^ observed that serum eosinophil cationic protein levels decreased in AD patients who were administered MA orally. Serum eosinophil cationic protein, a cytotoxic protein released by activated eosinophils, rises rapidly during acute exacerbations of AD and is considered to be an indicator of AD at acute exacerbations. Prakoeswa et al^35^ found that patients treated with MA plus TCS had lower levels of interleukin‐4 and interferon gamma and higher levels of interleukin‐10. Kim et al^53^ discovered that oral MA could reduce AD‐associated skin lesions, epidermal thickening, serum levels of immunoglobulin E, and immune cell infiltration. Therefore, we speculated that the therapeutic‐boosting effect of MA may be associated with the modulation of inflammatory factors and immunoreactive molecules. However, the specific mechanism of MA for AD is still unclear and needs to be further studied.

### 4.1 Implications for clinical practice

With regards to the types of MA, a mixture of probiotics (*Lactobacillus* and Bifidobacterium combined) was more effective than probiotics alone, which achieved the MCID in SCORAD index. Jiang et al.^54^ and Uwaezuoke et al.^55^ discovered that probiotics could relieve AD symptoms, especially using mixed‐strain probiotics (*Lactobacillus* and Bifidobacterium). Another meta‐analysis^56^ also favored that mixed strains of *Lactobacillus* and Bifidobacterium could reduce the incidence of AD. Thus, a mixed‐strain probiotic component might be beneficial to patients with AD. The duration of MA ranged from 6 to 24 weeks, most of which were administered MA orally for 8 weeks or 12 weeks. Dosage form included tablets, liquids, powders, and capsules. The majority of the included studies reported that oral MA doses were 5–10 × 10^9^ colony‐forming units/day (CFU/day). CFU/day, a measure of the MA dose, was related to the positive effects if there were more than 10^8^ CFU/day.^57^ However, due to limited available studies, the clinical application of MA is not fully understood yet.^31^, ^42^ Future research should focus on the optimal clinical protocols of MA treatment for AD.

In addition, the results of subgroup analyses showed that MA ameliorated the symptoms of AD in children, but not in infants and adults. In contrast to adults, the gut microbiota of children was relatively unstable, which was vulnerable to external factors such as nutrition, diet and external environments.^58^, ^59^ Infants had a mono‐diet and their gut microbiota was mainly derived from breastfeeding or formula.^60^ Different feeding practices might affect the fixation of oral MA in the infant gut. Besides, oral MA supplementation had a therapeutic‐boosting effect in moderate or severe AD patients receiving TCS treatment. In patients with mild AD, the effect of MA could be masked by potent TCS. TCS can control the symptoms of most mild to moderate AD patients, but not severe AD patients.^6^MA as a complementary therapy to TCS treatment might be a promising treatment option for patients with moderate or severe AD.

We were also concerned about whether MA, as an adjunct therapy, had a steroid sparing effect. It would be beneficial for reducing the corticophobia and improving compliance with the therapeutic regimen. In the present study, no difference was detected in the frequency and grams of TCS after in combination with MA. Since few studies^42^, ^43^ focused on it, the result should be treated with caution. Meanwhile, due to limited studies, the steroid sparing effect in different ages is still unclear. More studies are needed to address this issue.

## 5 LIMITATIONS

Several potential limitations of the present meta‐analysis should be acknowledged. First, the number of included RCTs was limited, and the sample size of each study was small. Second, the relevant articles published in English were retrieved, and selection bias was inevitable. Third, owing to limited studies, the optimal protocols of MA plus TCS therapy were not determined (the optimal types of MA, the optimal dosage, frequency, et al.).

## 6 CONCLUSION

Oral MA plus TCS could be an effective and safe treatment for patients with AD to relieve relevant symptoms, which might be used as an add‐on therapy in the treatment of AD. Owing to limited available studies, results should be interpreted with caution. Further studies with a larger sample size are needed to explore the optimal protocol of MA plus TCS.

## AUTHOR CONTRIBUTIONS

Conceptualization, Peiwen Xue and Xianjun Xiao; Methodology, Juan Li and Rongjiang Jin; Software, Peiwen Xue; Validation, Xianjun Xiao, Juan Li and Rongjiang Jin; Formal Analysis, Juan Li and Rongjiang Jin; Investigation, Haiyan Qin and Di Qin; Resources, Peiwen Xue and Huilin Liu; Data Curation, Peiwen Xue, Haiyan Qin, Di Qin and Huilin Liu; Writing – Original Draft Preparation, Peiwen Xue; Writing – Review & Editing, Peiwen Xue and Xianjun Xiao; Supervision, Rongjiang Jin and Juan Li; Project Administration, Peiwen Xue and Xianjun Xiao.

## CONFLICT OF INTEREST STATEMENT

The authors declare that they have no conflicts of interest.

## Supporting information

Supporting Information S1

Supporting Information S2

Supporting Information S3

## Data Availability

The datasets used and analyzed during the current study are available from the corresponding author upon reasonable request.
