# Supplementary material for: The efficacy and safety of oral microecological agents as add‐on therapy for atopic dermatitis: A systematic review and meta‐analysis of randomized clinical trials
Source: Clin Transl Allergy. 2023 Dec 4;13(12):e12318. doi: 10.1002/clt2.12318 (PMC10694634; doi:10.1002/clt2.12318)
Supplement: Supplementary file 1 — Supporting Information S1 [file CLT2-13-e12318-s003.docx]

**Ovid MEDLINE(R) and Epub Ahead of Print, In-Process, In-Data-Review & Other Non-Indexed Citations, Daily and Versions <1946 to April 07, 2023> 2023-4-11**

1 randomized controlled trial.pt. 590453

2 controlled clinical trial.pt. 95257

3 randomized.ab. 599061

4 trial.ti. 282784

5 placebo.ab. 237263

6 clinical trials as topic.sh. 200935

7 randomly.ab. 405774

8 1 or 2 or 3 or 4 or 5 or 6 or 7 1515403

9 exp animals/ not humans.sh. 5110173

10 8 not 9 1394537

11 exp Eczema/ or eczema.mp. 24582

12 neurodermatitis.mp. or exp Neurodermatitis/ 1809

13 exp Dermatitis, Atopic/ 24194

14 exp Dermatitis/ or dermatitis.mp. 137591

15 besnier$ prurigo.mp. 49

16 11 or 12 or 13 or 14 or 15 142652

17 exp Probiotics/ 24057

18 probiotic$.mp. 41099

19 exp Lactobacillus/ 24509

20 lactobacill$.mp. 51941

21 exp Bifidobacterium/ 7203

22 bifidobacteri$.mp. 15275

23 exp Lactococcus/ 5614

24 lactococc$.mp. 9263

25 exp Saccharomyces/ or saccharomyces.mp. 147720

26 streptococcus thermophilus.mp. or exp Streptococcus thermophilus/ 2286

27 lactic acid bacteri$.mp. 14896

28 bacillus subtilis.mp. or exp Bacillus subtilis/ 41035

29 enterococcus faecalis.mp. or exp Enterococcus faecalis/ 18660

30 exp Prebiotics/ 4763

31 prebiotic$.mp. 13277

32 exp Synbiotics/ 1068

33 synbiotic$.mp. 2457

34 17 or 18 or 19 or 20 or 21 or 22 or 23 or 24 or 25 or 26 or 27 or 28 or 29 or 30 or 31 or 32 or 33 299418

35 prurit$ dermatoses.mp. 155

36 prurit$ skin disease$.mp. 161

37 itch$ dermatoses.mp. 50

38 itch$ skin disease$.mp. 36

39 35 or 36 or 37 or 38 400

40 16 or 39 142843

41 10 and 34 and 40 349

**Embase <1974 to 2023 April 07> 2023-4-11**

1 randomized controlled trial.sh. 778157

2 controlled clinical trial.sh. 468927

3 random$.tw. 1949832

4 trial.ti. 398801

5 placebo$.tw. 365747

6 1 or 2 or 3 or 4 or 5 2427920

7 exp animal/ or exp invertebrate/ or animal experiment/ or animal model/ or animal tissue/ or animal cell/ or nonhuman/ 32551602

8 human/ or normal human/ 25308280

9 7 and 8 25308280

10 7 not 9 7243322

11 6 not 10 2174737

12 eczema.mp. or exp ECZEMA/ 48741

13 exp DERMATITIS/ or dermatitis.mp. 207594

14 exp atopic dermatitis/ 55928

15 neurodermatitis.mp. or exp NEURODERMATITIS/ 3500

16 besnier$ prurigo.mp. 19

17 12 or 13 or 14 or 15 or 16 210686

18 exp probiotic agent/ 51776

19 probiotic$.mp. 61327

20 exp Lactobacillus/ 60902

21 lactobacill$.mp. 70863

22 exp Bifidobacterium/ 20532

23 bifidobacteri$.mp. 26426

24 exp Lactococcus/ 9707

25 lactococc$.mp. 11764

26 Saccharomyces.mp. or exp Saccharomyces/ 125577

27 Streptococcus thermophilus.mp. or exp Streptococcus thermophilus/ 4011

28 lactic acid bacteri$.mp. 17554

29 Bacillus subtilis.mp. or exp Bacillus subtilis/ 55100

30 Enterococcus faecalis.mp. or exp Enterococcus faecalis/ 32507

31 exp Prebiotics/ 12242

32 prebiotic$.mp. 18591

33 exp Synbiotics/ 2876

34 synbiotic$.mp. 3706

35 18 or 19 or 20 or 21 or 22 or 23 or 24 or 25 or 26 or 27 or 28 or 29 or 30 or 31 or 32 or 33 or 34 333013

36 prurit$ dermatoses.mp. 154

37 prurit$ skin disease$.mp. 275

38 itch$ dermatoses.mp. 44

39 itch$ skin disease$.mp. 74

40 36 or 37 or 38 or 39 540

41 17 or 40 210855

42 11 and 35 and 41 643

**APA PsycInfo <1806 to April Week 1 2023> 2023-4-11**

1 random$ assigned.tw. 41185

2 control.tw. 501354

3 double blind.tw. 24816

4 1 or 2 or 3 544294

5 eczema.ti,ab. or exp Eczema/ 452

6 dermatitis.ti,ab. or exp Dermatitis/ 974

7 neurodermatitis.ti,ab. or exp Neurodermatitis/ 96

8 besnier$ prurigo.mp. 1

9 5 or 6 or 7 or 8 1246

10 probiotic$.mp. 556

11 (lactobacill$ or bifidobacteri$ or lactococc$ or saccharomyces or microbiome$ or microbiotica).mp. 1740

12 lactic acid bacteri$.mp. 18

13 bacillus subtilis.mp. 18

14 streptococcus thermophilus.mp. 1

15 enterococcus faecalis.mp. 15

16 prebiotics.ti,ab. or exp Prebiotics/ 117

17 synbiotics.ti,ab. or exp Synbiotics/ 11

18 prebiotic$.mp. 172

19 synbiotic$.mp. 23

20 10 or 11 or 12 or 13 or 14 or 15 or 16 or 17 or 18 or 19 2066

21 prurit$ dermatoses.mp. 2

22 prurit$ skin disease$.mp. 5

23 itch$ dermatoses.mp. 1

24 itch$ skin disease$.mp. 2

25 21 or 22 or 23 or 24 10

26 9 or 25 1252

27 4 and 20 and 26 2

**Cochrane Library (2023-4-11)**

Search Name:

Date Run: 11/04/2023 04:07:15

Comment:

ID Search Hits

#1 (probiotic*):ti,ab,kw OR (prebiotic*):ti,ab,kw OR (Synbiotic*):ti,ab,kw OR (Lactobacill*):ti,ab,kw OR (Bifidobacteri*):ti,ab,kw OR (Lactococc*):ti,ab,kw OR (Streptococc*):ti,ab,kw OR (saccharomyces):ti,ab,kw OR (probiotics):ti,ab,kw OR (prebiotics):ti,ab,kw OR (Synbiotics):ti,ab,kw 18806

#2 (Atopic Dermatitides):ti,ab,kw OR (Atopic Dermatitis):ti,ab,kw OR (Dermatitides, Atopic):ti,ab,kw OR (Eczema, Atopic):ti,ab,kw OR (Atopic Eczema):ti,ab,kw OR (Eczema, Infantile):ti,ab,kw OR (Infantile Eczema):ti,ab,kw OR (Dermatitis, Atopic):ti,ab,kw 6031

#3 ((prurit* dermatoses) OR (prurit* skin disease) OR (itch* dermatoses) OR (itch* skin disease)):ti,ab,kw 2687

#4 (#2 or #3) 8164

#5 (randomized controlled trial):ti,ab,kw OR (controlled clinical trial):ti,ab,kw OR (randomized):ti,ab,kw OR (placebo):ti,ab,kw OR (clinical trials as topic):ti,ab,kw OR (randomly):ti,ab,kw 1332521

#6 (#1 and #4 and #5) 398

**Web of Science 2023-4-11**

1: TS=(Atopic Dermatitides or Atopic Dermatitis or Dermatitides, Atopic or Eczema, Atopic or Atopic Eczema or Eczema, Infantile or Infantile Eczema or Dermatitis, Atopic) 31499

2: TS=(prurit* skin disease* or itch* skin disease* or itch* dermatoses or prurit* dermatoses) 9782

3: TS=(probiotic* or prebiotic* or Synbiotic* or Lactobacill* or Bifidobacteri* or Lactococc* or Streptococc* or saccharomyces or probiotics or prebiotics or Synbiotics ) 253480

4: TS=(clinical trial* or research design or comparative stud* or evaluation stud* or controlled trial* or follow-up stud* or prospective stud* or random* or random* or single blind* or double blind* ) 5846821

5: (#2 OR #1) 8568

6: (#5 AND #3 AND #4) 758
