# Supplementary material for: The efficacy and safety of oral microecological agents as add‐on therapy for atopic dermatitis: A systematic review and meta‐analysis of randomized clinical trials
Source: Clin Transl Allergy. 2023 Dec 4;13(12):e12318. doi: 10.1002/clt2.12318 (PMC10694634; doi:10.1002/clt2.12318)
Supplement: Supplementary file 2 — Supporting Information S2 [file CLT2-13-e12318-s001.docx]

**Table: The excluded studies with the reasons for exclusion**

| **No.** | **Study/year** | **Title** | **Excluded Reason** |
| --- | --- | --- | --- |
| 1 | Actrn  2007 | A Phase 1/2 Single Center, Randomized, Double-blind, Placebo-controlled, Left-Right Comparison Study to Evaluate the Safety and Preliminary Efficacy of a Topical Antimicrobial (NEO101) in Older Adolescents and Adults with at least Moderate Atopic Dermatitis | 1 |
| 2 | Ahn  2020 | Effects of Lactobacillus pentosus in Children with Allergen-Sensitized Atopic Dermatitis | 1 |
| 3 | Astari  2020 | Evaluation of scorad in adults with atopic dermatitis receiving lactobacillus plantarum is-10506 supplementation | 1 |
| 4 | Axt-Gadermann  2018 | Significant improvement of skin conditions in atopic dermatitis by synbiotic bath additives | 1 |
| 5 | Axt-Gadermann  2021 | Probiotic Partial Baths for atopic Dermatitis | 1 |
| 6 | Bozensky  2015 | Prebiotics do not influence the severity of atopic dermatitis in infants: A randomised controlled trial | 1 |
| 7 | Brouwer  2006 | No effects of probiotics on atopic dermatitis in infancy: a randomized placebo-controlled trial | 1 |
| 8 | Butler  2020 | Lactobacillus reuteriDSM 17938 as a Novel Topical Cosmetic Ingredient: A Proof of Concept Clinical Study in Adults with Atopic Dermatitis | 1 |
| 9 | Carucci  2022 | Therapeutic effects elicited by the probiotic Lacticaseibacillus rhamnosus GG in children with atopic dermatitis. The results of the ProPAD trial | 1 |
| 10 | Chaiyasut  2022 | Influence of Paraprobiotics-Containing Moisturizer on Skin Hydration and Microbiome: A Preliminary Study | 1 |
| 11 | Chernyshov  2009 | Randomized, placebo-controlled trial on clinical and immunologic effects of probiotic containing Lactobacillus rhamnosus R0011 and L. helveticus R0052 in infants with atopic dermatitis | 1 |
| 12 | ChiCtr  2018 | Effects of probiotics on inflammation and intestinal microflora in patients with atopic dermatitis | 1 |
| 13 | ChiCtr  2019 | Effects of probiotics (CCFM1029) on inflammation and intestinal microflora in patients with atopic dermatitis | 1 |
| 14 | ChiCtr  2021 | Effect of Bifidobacterium lactis oil drops on the management of eczema in infants: a randomized double-blind parallel contrast study | 1 |
| 15 | Climent  2021 | Changes in Gut Microbiota Correlates with Response to Treatment with Probiotics in Patients with Atopic Dermatitis. A Post Hoc Analysis of a Clinical Trial | 1 |
| 16 | Cukrowska  2010 | Probiotic lactobacillus casei and lactobacillus paracasei strains in treatment of food allergy in children | 1 |
| 17 | Cukrowska  2021 | The Effectiveness of Probiotic Lactobacillus rhamnosus and Lactobacillus casei Strains in Children with Atopic Dermatitis and Cow's Milk Protein Allergy: a Multicenter, Randomized, Double Blind, Placebo Controlled Study | 1 |
| 18 | Cukrowska  2008 | The influence of probiotic Lactobacillus casei and paracasei strains on clinical status of atopic eczema in children with food allergy on cow's milk proteins | 1 |
| 19 | Dissanayake  2019 | Skin Care and Synbiotics for Prevention of Atopic Dermatitis or Food Allergy in Newborn Infants: a 2 × 2 Factorial, Randomized, Non-Treatment Controlled Trial | 1 |
| 20 | Drago  2014 | Treatment of atopic dermatitis eczema with a high concentration of Lactobacillus salivarius LS01 associated with an innovative gelling complex a pilot study on adults | 1 |
| 21 | Drago  2011 | Effects of Lactobacillus salivarius LS01 (DSM 22775) treatment on adult atopic dermatitis: A randomized placebo-controlled study | 1 |
| 22 | Euctr  2006 | Probiotics to infants with atopic dermatitis; an investigation of the effect on eczema, immune system and intestinal microflora, inflammation and permeability. DK-titel Probiotiske bakterier til børn med atopisk dermatitis; undersøgelse af effekten på eksem, immunstatus, intestinal mikroflora samt tarmens inflammation og permeabilitet | 1 |
| 23 | Fang  2020 | Probiotics modulate the gut microbiota composition and immune responses in patients with atopic dermatitis: a pilot study | 1 |
| 24 | Farid  2011 | Effect of a new synbiotic mixture on atopic dermatitis in children: A randomized-controlled trial | 1 |
| 25 | Farid  2010 | Clinical and immunogical effect of probiotic in childhood atopic dermatitis | 1 |
| 26 | Ghanei  2014 | Objective score of atopic dermatitis and prebiotic in infant | 1 |
| 27 | Ghanei  2011 | Effectiveness of prebiotic in atopic dermatitis reduction in 7-24 months old children living in Isfahan | 1 |
| 28 | Gobel  2010 | Probiotics to young children with atopic dermatitis: A randomized placebo-controlled trial | 1 |
| 29 | Gore  2012 | Treatment and secondary prevention effects of the probiotics Lactobacillus paracasei or Bifidobacterium lactis on early infant eczema: randomized controlled trial with follow-up until age 3 years | 1 |
| 30 | Grueber  2015 | Immunoactive prebiotics transiently prevent occurrence of early atopic dermatitis among low- atopy- risk infants | 1 |
| 31 | Herwanto  2020 | A randomized controlled trial of lactobacillus plantarum is-10506 supplementation for atopic dermatitis in adults: Effects on quality of life | 1 |
| 32 | Hubbard  2022 | Synbiotic containing extensively hydrolyzed formula improves gastrointestinal and atopic symptom severity, growth, caregiver quality of life, and hospital-related healthcare use in infants with cow's milk allergy | 1 |
| 33 | Hulshof  2017 | Dietary intervention with a synbiotic mixture of scGOS/lcFOS with bifidobacterium breve M-16V in infants with atopic dermatitis shows beneficial immunological changes in chemokine profiles | 1 |
| 34 | Ido  2018 | The effect of prebiotic lactosucrose on serum ige levels in allergic people: A pilot study in Japan | 1 |
| 35 | Iemoli  2012 | Probiotics reduce gut microbial translocation and improve adult atopic dermatitis | 1 |
| 36 | Irct201010204976N  2011 | Synbiotic in Atopic Dermatitis in children | 1 |
| 37 | Irct20120215009014N  2019 | Effect of topical product including Lactobacillus lysate versus placebo on the clinical symptoms in the patients with atopic dermatitis | 1 |
| 38 | Irct20170513033941N  2019 | Combined probiotic and melatonin supplementation in treatment of atopic dermatitis | 1 |
| 39 | Isolauri  2000 | Probiotics in the management of atopic eczema | 1 |
| 40 | Isrctn  2005 | Probiotics in Atopic Dermatitis in Infancy | 1 |
| 41 | Isrctn  2005 | Pilot study: probiotics in children with atopic dermatitis and food allergy - is there an in vivo or in vitro effect on the immunological response? | 1 |
| 42 | Isrctn  2020 | Is a mixture of selected probiotic strains effective in improving eczema related symptoms? | 1 |
| 43 | Jeong  2020 | A randomized trial of Lactobacillus rhamnosus IDCC 3201 tyndallizate (RHT3201) for treating atopic dermatitis | 1 |
| 44 | Kaur  2008 | Successful management of mild atopic dermatitis in adults with probiotics and emollients | 1 |
| 45 | Kct  2019 | Clinical trial for the evaluation of the efficacy and safety of live or killed Lactobacillus sakei Probio65 on atopic dermatitis in children | 1 |
| 46 | Kct  2021 | A 12 week, randomized, double-blind, placebo-controlled clinical trial for the evaluation of the efficacy and safety of KBL693 on atopic dermatitis in children | 1 |
| 47 | Kct  2021 | Efficacy and Safety of Advanced Fructo-oligosaccharide (FOS) on Atopic Dermatitis: clinical trial | 1 |
| 48 | Kim  2017 | Identification of atopic dermatitis phenotypes with good responses to probiotics (Lactobacillus plantarum CJLP133) in children | 1 |
| 49 | Lin  2015 | Protective effect of probiotics in the treatment of infantile eczema | 1 |
| 50 | Matsumoto  2007 | LKM512 yogurt consumption improves the intestinal environment and induces the T-helper type 1 cytokine in adult patients with intractable atopic dermatitis | 1 |
| 51 | Michelotti  2021 | Efficacy of a probiotic supplement in patients with atopic dermatitis: a randomized, double-blind, placebo-controlled clinical trial | 1 |
| 52 | Murosaki  2006 | Effects of intake of syrup supplemented with nigerooligosaccharides and heat-killed Lactobacillus plantarum L-137 on skin symptom and immune function in patients with atopic dermatitis | 1 |
| 53 | Nakata  2019 | Additive effect of Lactobacillus acidophilus L-92 on children with atopic dermatitis concomitant with food allergy | 1 |
| 54 | Nct  2005 | Double-Blind, Parallel, Randomised Study to Investigate the Effect of Oral Probiotics in Infants With Atopic Dermatitis | 1 |
| 55 | Nct  2006 | The Effects of Probiotics in Atopic Dermatitis | 1 |
| 56 | Nct  2009 | Probiotic Bacteria to Infants With Atopic Dermatitis | 1 |
| 57 | Nct  2010 | Effect of Probiotics in the Atopic Dermatitis | 1 |
| 58 | Nct  2011 | Probiotics in Adults: do They Improve Atopic Dermatitis? | 1 |
| 59 | Nct  2014 | Assessment of Efficacy of the Consumption of a Heat-treated Lactobacillus Paracasei (GM080) on Atopic Dermatitis | 1 |
| 60 | Nct  2016 | Effects of Lactobacillus Reuteri Plus Vitamin D3 in Children With Atopic Dermatitis | 1 |
| 61 | Nct  2020 | A Study of a Probiotic Food Supplement Containing B. Infantis (EVC001) in Healthy Breastfed Infants at Risk of Developing Atopic Dermatitis | 1 |
| 62 | Nct  2021 | Study of the Skin Microbiome and the Potential of a Topical Probiotic Cream for Atopic Dermatitis | 1 |
| 63 | Nct  2022 | Probiotic on Atopic Dermatitis in Infant | 1 |
| 64 | Nct  2023 | The Efficacy of NTU 101 Lactic Acid Bacteria Powder in the Adjuvant Improvement of Atopic Dermatitis Clinical Trial | 1 |
| 65 | Nermes  2009 | Probiotics promote the development of memory B cells in infants with atopic dermatitis | 1 |
| 66 | Nermes  2011 | Interaction of orally administered Lactobacillus rhamnosus GG with skin and gut microbiota and humoral immunity in infants with atopic dermatitis | 1 |
| 67 | Niccoli  2014 | Preliminary Results on Clinical Effects of Probiotic Lactobacillus salivarius LS01 in Children Affected by Atopic Dermatitis | 1 |
| 68 | Noll  2021 | Improvement of Atopic Dermatitis by Synbiotic Baths | 1 |
| 69 | Park  2014 | Effect of Emollients Containing Vegetable-Derived Lactobacillus in the Treatment of Atopic Dermatitis Symptoms: split-Body Clinical Trial | 1 |
| 70 | Prakoeswa  2022 | Beneficial effect of Lactobacillus plantarum IS-10506 supplementation in adults with atopic dermatitis: a randomized controlled trial | 1 |
| 71 | Prakoeswa  2020 | The beneficial effect of lactobacillus plantarum is-10506 supplementation in adults with atopic dermatitis: A randomized control trial | 1 |
| 72 | Prescott  2005 | Clinical effects of probiotics are associated with increased interferon-gamma responses in very young children with atopic dermatitis | 1 |
| 73 | Rather  2021 | Oral Administration of Live and Dead Cells of Lactobacillus sakei proBio65 Alleviated Atopic Dermatitis in Children and Adolescents: a Randomized, Double-Blind, and Placebo-Controlled Study | 1 |
| 74 | Rosenfeldt  2004 | Effect of probiotics on gastrointestinal symptoms and small intestinal permeability in children with atopic dermatitis | 1 |
| 75 | Torii  2011 | Effects of oral administration of Lactobacillus acidophilus l-92 on the symptoms and serum markers of atopic dermatitis in children | 1 |
| 76 | Umin  2011 | A randomized, open-label, parallel group study to evaluate the efficacy and safety of the oral administration of Bifidobacterium animalis subsp. lactis LKM512 in patients with atopic dermatitis | 1 |
| 77 | Umin  2013 | Skin care and synbiotics for prevention of atopic dermatitis or food allergy in newborn infants: a 2 x 2 factorial, randomized, non-treatment controlled trial | 1 |
| 78 | Van  2009 | Effect of a new synbiotic mixture on atopic dermatitis in infants: A randomised controlled trial | 1 |
| 79 | Viljanen  2005 | Probiotics in the treatment of atopic eczema/dermatitis syndrome in infants: A double-blind placebo-controlled trial | 1 |
| 80 | Wang  2015 | Children with atopic dermatitis show clinical improvement after Lactobacillus exposure | 1 |
| 81 | Wang  2022 | Effect of a Novel E3 Probiotics Formula on the Gut Microbiome in Atopic Dermatitis Patients: A Pilot Study | 1 |
| 82 | Weston  2005 | Probiotics provide clinical benefit in moderate and severe atopic dermatitis: a randomised controlled trial | 1 |
| 83 | Wickens  2011 | A differential effect of 2 probiotics on allergic disease to age 4 years | 1 |
| 84 | Yang  2014 | Efficacy of probiotic therapy on atopic dermatitis in children: A randomized, double-blind, placebo-controlled trial | 1 |
| 85 | Yesilova  2012 | Effect of probiotics on the treatment of children with atopic dermatitis | 1 |
| 86 | Yim  2006 | Therapeutic Effects of Probiotics in Patients with Atopic Dermatitis | 1 |
| 87 | Yoshida  2010 | Clinical effects of probiotic bifidobacterium breve supplementation in adult patients with atopic dermatitis | 1 |
| 88 | Bodemer  2017 | Adjuvant treatment with the bacterial lysate (OM-85) improves management of atopic dermatitis: A randomized study | 1 |
| 89 | Seite  2017 | Clinical efficacy of emollients in atopic dermatitis patients - relationship with the skin microbiota modification | 1 |
| 90 | Passeron  2006 | Prebiotics and synbiotics: Two promising approaches for the treatment of atopic dermatitis in children above 2 years | 2 |
| 91 | Wu  2012 | Lactobacillus salivarius plus fructo-oligosaccharide is superior to fructo-oligosaccharide alone for treating children with moderate to severe atopic dermatitis: a double-blind, randomized, clinical trial of efficacy and safety | 2 |
| 92 | Hattori  2003 | Effects of administration of Bifidobacteria on fecal microflora and clinical symptoms in infants with atopic dermatitis | 3 |
| 93 | Ismail  2016 | Early gut colonization by Bifidobacterium breve and B. catenulatum differentially modulates eczema risk in children at high risk of developing allergic disease | 3 |
| 94 | Ismail  2014 | Reduced neonatal regulatory T cell response to microbial stimuli associates with subsequent eczema in high-risk infants | 3 |
| 95 | Kauppi  2014 | Interaction of NPSR1 genotypes and probiotics in the manifestation of atopic eczema in early childhood | 3 |
| 96 | Kim  2015 | Probiotic supplementation influences faecal short chain fatty acids in infants at high risk for eczema | 3 |
| 97 | Kingkaw  2020 | Analysis of the infant gut microbiome reveals metabolic functional roles associated with healthy infants and infants with atopic dermatitis using metaproteomics | 3 |
| 98 | Kirjavainen  2002 | Aberrant composition of gut microbiota of allergic infants: A target of bifidobacterial therapy at weaning? | 3 |
| 99 | Klewicka  2011 | Changes in gut microbiota in children with atopic dermatitis administered the bacteria Lactobacillus casei DN - 114001 | 3 |
| 100 | Kosuwon  2018 | A synbiotic mixture of scGOS/lcFOS and Bifidobacterium breve M-16V increases faecal Bifidobacterium in healthy young children | 3 |
| 101 | Low  2017 | Ratio of Klebsiella/Bifidobacterium in early life correlates with later development of paediatric allergy | 3 |
| 102 | Matsumoto  2007 | LKM512 yogurt consumption improves the intestinal environment and induces the T-helper type 1 cytokine in adult patients with intractable atopic dermatitis | 3 |
| 103 | Miniello  2010 | Lactobacillus reuteri modulates cytokines production in exhaled breath condensate of children with atopic dermatitis | 3 |
| 104 | Murosaki  2006 | Effects of intake of syrup supplemented with nigerooligosaccharides and heat-killed Lactobacillus plantarum L-137 on skin symptom and immune function in patients with atopic dermatitis | 3 |
| 105 | Nct  2022 | Probiotic on Atopic Dermatitis in Infant | 3 |
| 106 | Nermes  2009 | Probiotics promote the development of memory B cells in infants with atopic dermatitis | 3 |
| 107 | Nermes  2011 | Interaction of orally administered Lactobacillus rhamnosus GG with skin and gut microbiota and humoral immunity in infants with atopic dermatitis | 3 |
| 108 | Park  2014 | Effect of Emollients Containing Vegetable-Derived Lactobacillus in the Treatment of Atopic Dermatitis Symptoms: split-Body Clinical Trial | 3 |
| 109 | Plaza  2022 | Effects of a novel infant formula on weight gain and body composition of infants: The INNOVA 2020 study | 3 |
| 110 | Prescott  2005 | Clinical effects of probiotics are associated with increased interferon-gamma responses in very young children with atopic dermatitis | 3 |
| 111 | Roessler  2012 | The effect of probiotics on faecal microbiota and genotoxic activity of faecal water in patients with atopic dermatitis: a randomized, placebo-controlled study | 3 |
| 112 | Rosenfeldt  2004 | Effect of probiotics on gastrointestinal symptoms and small intestinal permeability in children with atopic dermatitis | 3 |
| 113 | Scalabrin  2017 | Long-term safety assessment in children who received hydrolyzed protein formulas with Lactobacillus rhamnosus GG: a 5-year follow-up | 3 |
| 114 | Takahashi  2019 | Effects of Probiotic Supplementation on TGF-beta 1, TGF-beta 2, and IgA Levels in the Milk of Japanese Women: An Open-Label Pilot Study | 3 |
| 115 | Taniuchi  2005 | Administration of Bifidobacterium to infants with atopic dermatitis: Changes in fecal microflora and clinical symptoms | 3 |
| 116 | Taylor  2007 | FOXP3 mRNA expression at 6 months of age is higher in infants who develop atopic dermatitis, but is not affected by giving probiotics from birth | 3 |
| 117 | Van  2009 | Beneficial effects of a synbiotic mixture on the intestinal microbiota of infants with atopic dermatitis | 3 |
| 118 | Van  2012 | No detectable beneficial systemic immunomodulatory effects of a specific synbiotic mixture in infants with atopic dermatitis | 3 |
| 119 | Viljanen  2005 | Probiotic effects on faecal inflammatory markers and on faecal IgA in food allergic atopic eczema/dermatitis syndrome infants | 3 |
| 120 | Wang  2022 | Effect of a Novel E3 Probiotics Formula on the Gut Microbiome in Atopic Dermatitis Patients: A Pilot Study | 3 |
| 121 | Wopereis  2018 | Intestinal microbiota in infants at high risk for allergy: Effects of prebiotics and role in eczema development | 3 |
| 122 | Guo  2015 | Clinical effect of microecologics as an adjuvant therapy on infants' eczema | 4 |
| 123 | Actrn  2016 | The SynAD study aims to determine if daily treatment with an oral preparation containing healthy bacteria, given together with specific carbohydrates to help them thrive, is effective in reducing severity of atopic dermatitis (AD) in food allergic children aged between 6 months and 3 years | 5 |
| 124 | Bisyuk  2023 | Combinations of Probiotic Bacteria (Lactobacillus helveticus, Bifidobacterium longum, Lactobacillus rhamnosus, Sacchammyces boulardii) Restores the Skin Microbiome in Atopic Dermatitis | 5 |
| 125 | Jaramillo  2013 | Probiotics in primary prevention of atopic dermatitis in infants at risk of suffering it | 5 |
| 126 | Perry  2006 | Effects of Probiotics on Atopic Dermatitis: A Randomised Controlled Trial | 5 |
| 127 | Zaynullina  2020 | Efficacy of a multicomponent probiotic in comprehensive therapy for atopic dermatitis in children | 5 |
| 128 | Zhang  2018 | The Clinical Observation of Bifid Triple Viable Powder Combined with Hydrocortisone Butyrate Ointment in Treating Infantile Eczema | 5 |
| 129 | CTRI/2017/08/009236  2017 | A study to observe if probiotics supplementation is helpful in atopic dermatitis children | 6 |
| 130 | CTRI/2017/10/010018  2017 | Probiotics in the treatment of atopic dermatitis in children | 6 |
| 131 | IRCT20101020004976N8  2021 | The effect of topical probiotic lotion on atopic dermatitis in children | 6 |
| 132 | NCT03822624  2019 | Study to Evaluate the Use of a Probiotic in SCORAD Reduction in Young Patients With Atopic Dermatitis | 6 |
| 133 | NCT04706559  2021 | Efficacy of Oral Supplementation of Probiotics in Children With Atopic Dermatitis | 6 |

Reason 1: Ineligible interventions

Reason 2: Ineligible controls

Reason 3: Irrelevant outcomes

Reason 4: Language non-English

Reason 5: Non-full text

Reason 6: Still being researched
