# Supplementary material for: The efficacy and safety of oral microecological agents as add‐on therapy for atopic dermatitis: A systematic review and meta‐analysis of randomized clinical trials
Source: Clin Transl Allergy. 2023 Dec 4;13(12):e12318. doi: 10.1002/clt2.12318 (PMC10694634; doi:10.1002/clt2.12318)
Supplement: Supplementary file 3 — Supporting Information S3 [file CLT2-13-e12318-s002.docx]

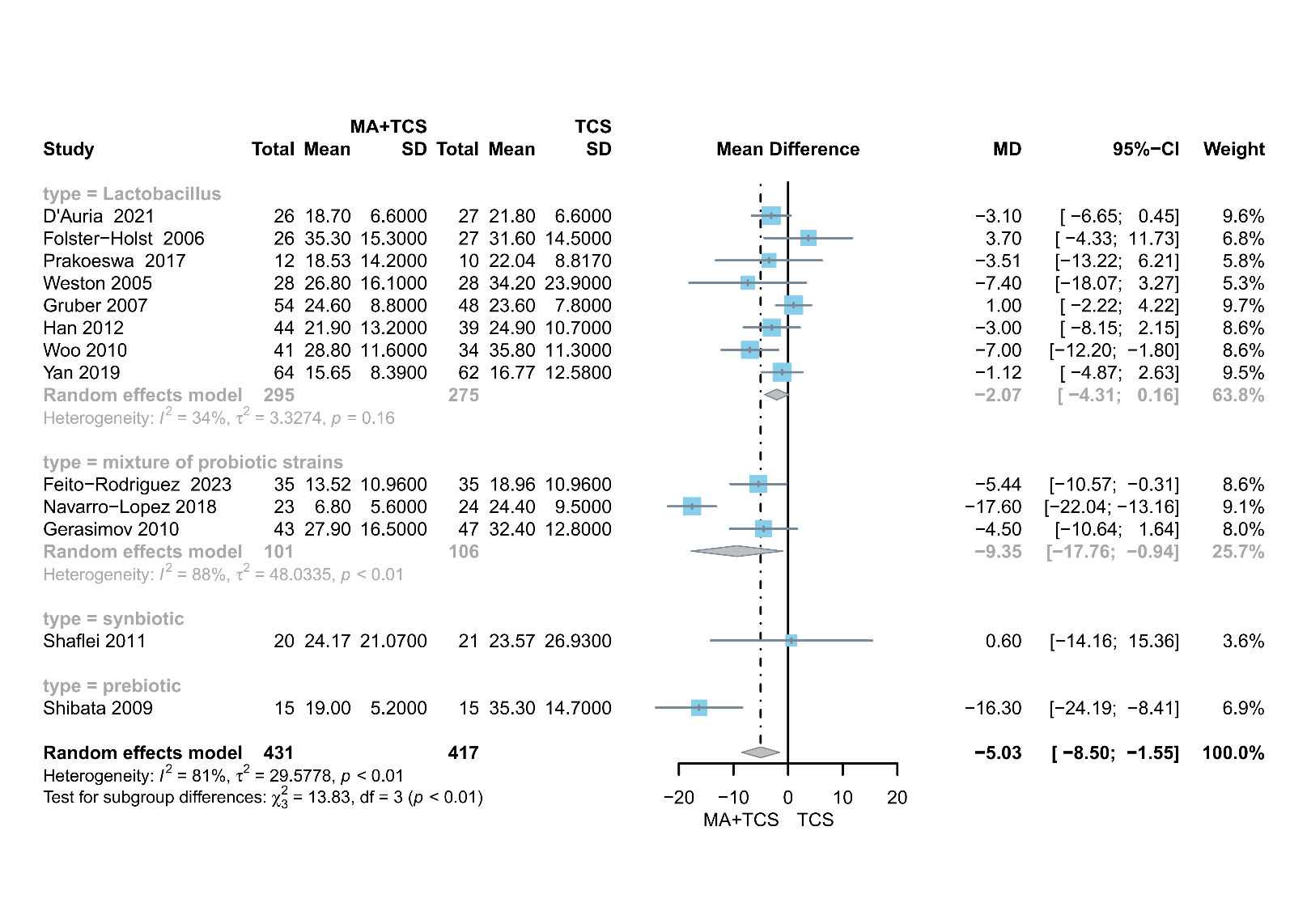


Figure S1 Forest plot of SCORSD scores (subgroup by types)


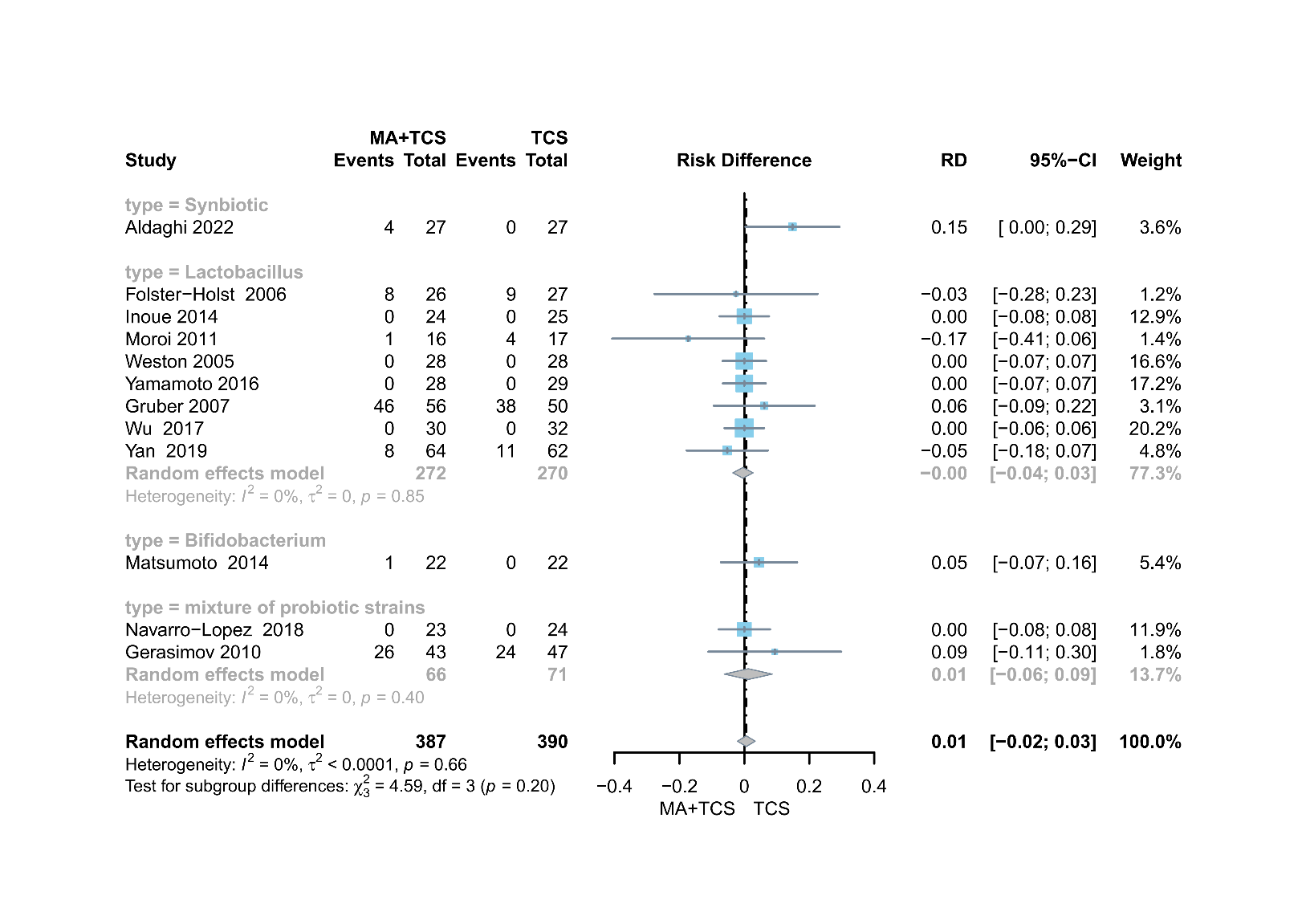


Figure S2 Forest plot of adverse events (subgroup by types)


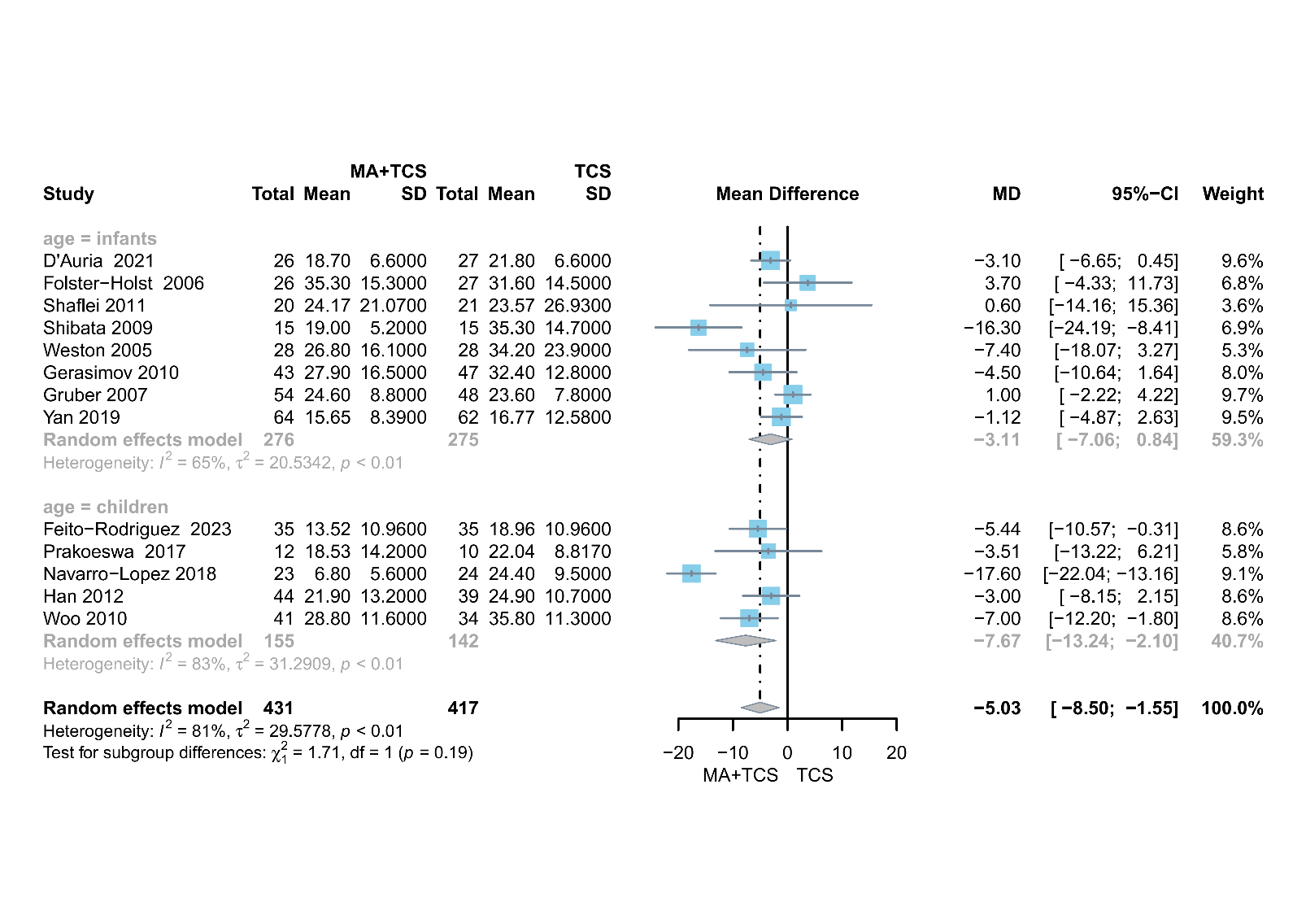


Figure S3 Forest plot of SCORSD scores (subgroup by ages)


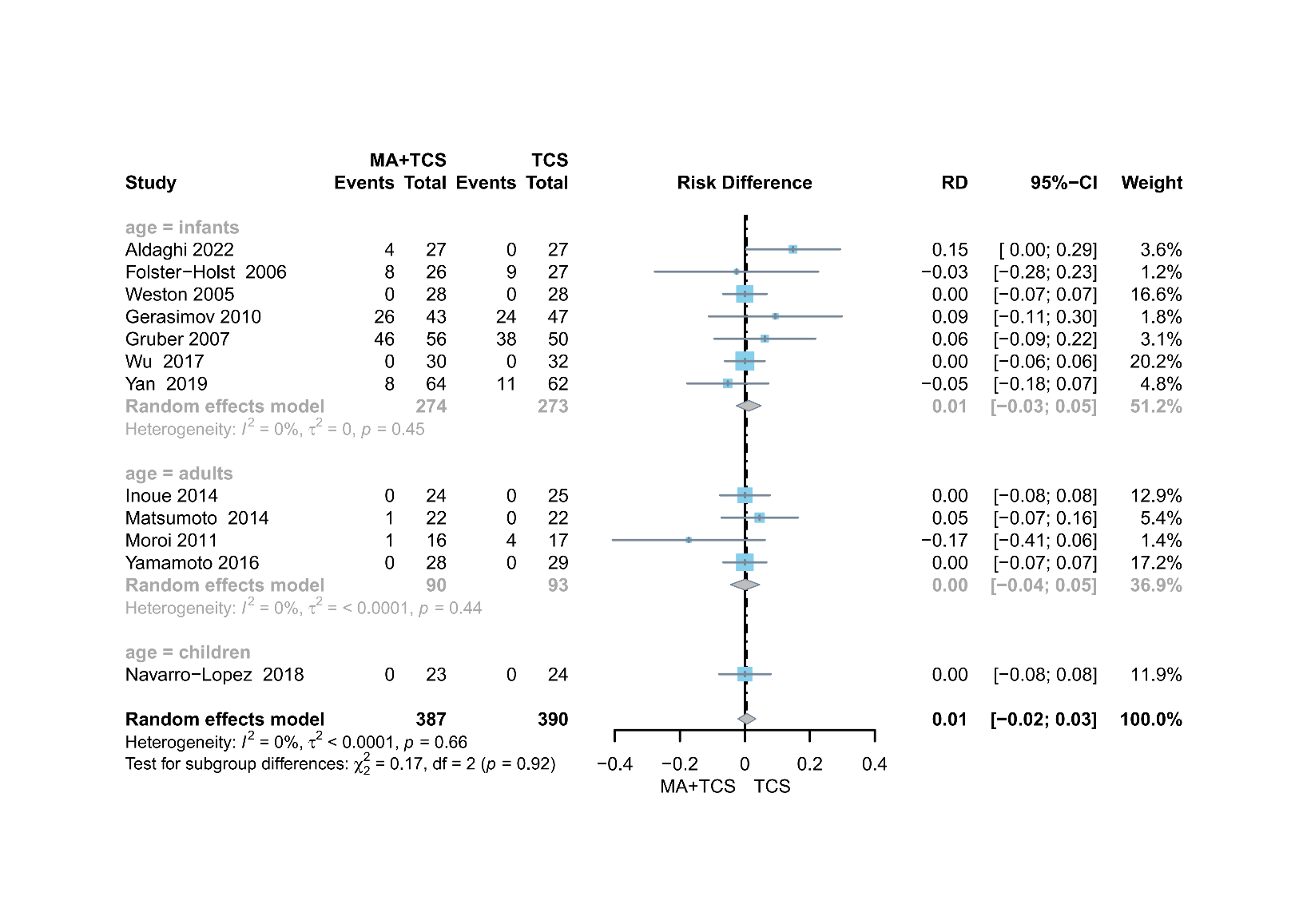


Figure S4 Forest plot of adverse events (subgroup by ages)


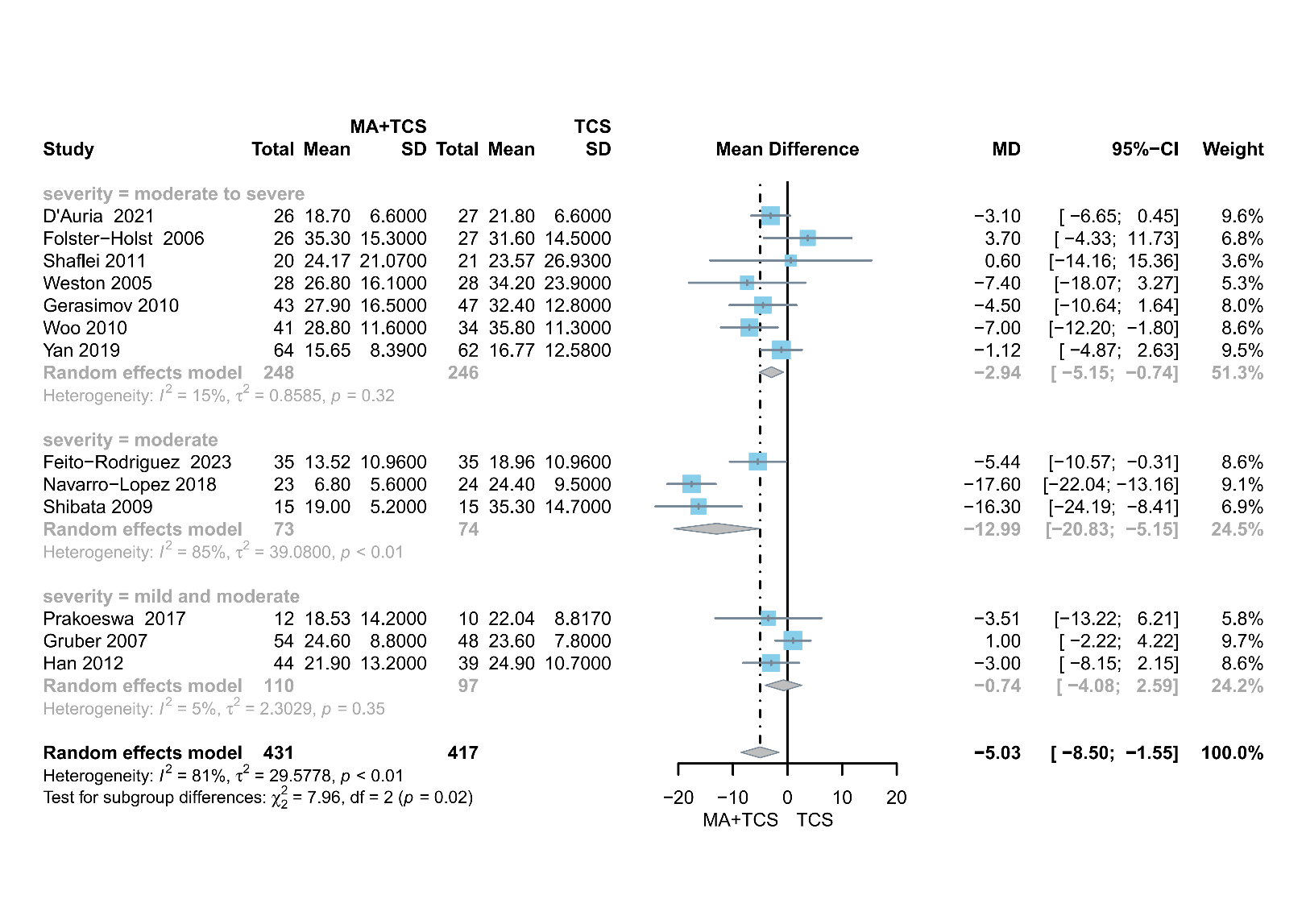


Figure S5 Forest plot of SCORSD scores (subgroup by severities)


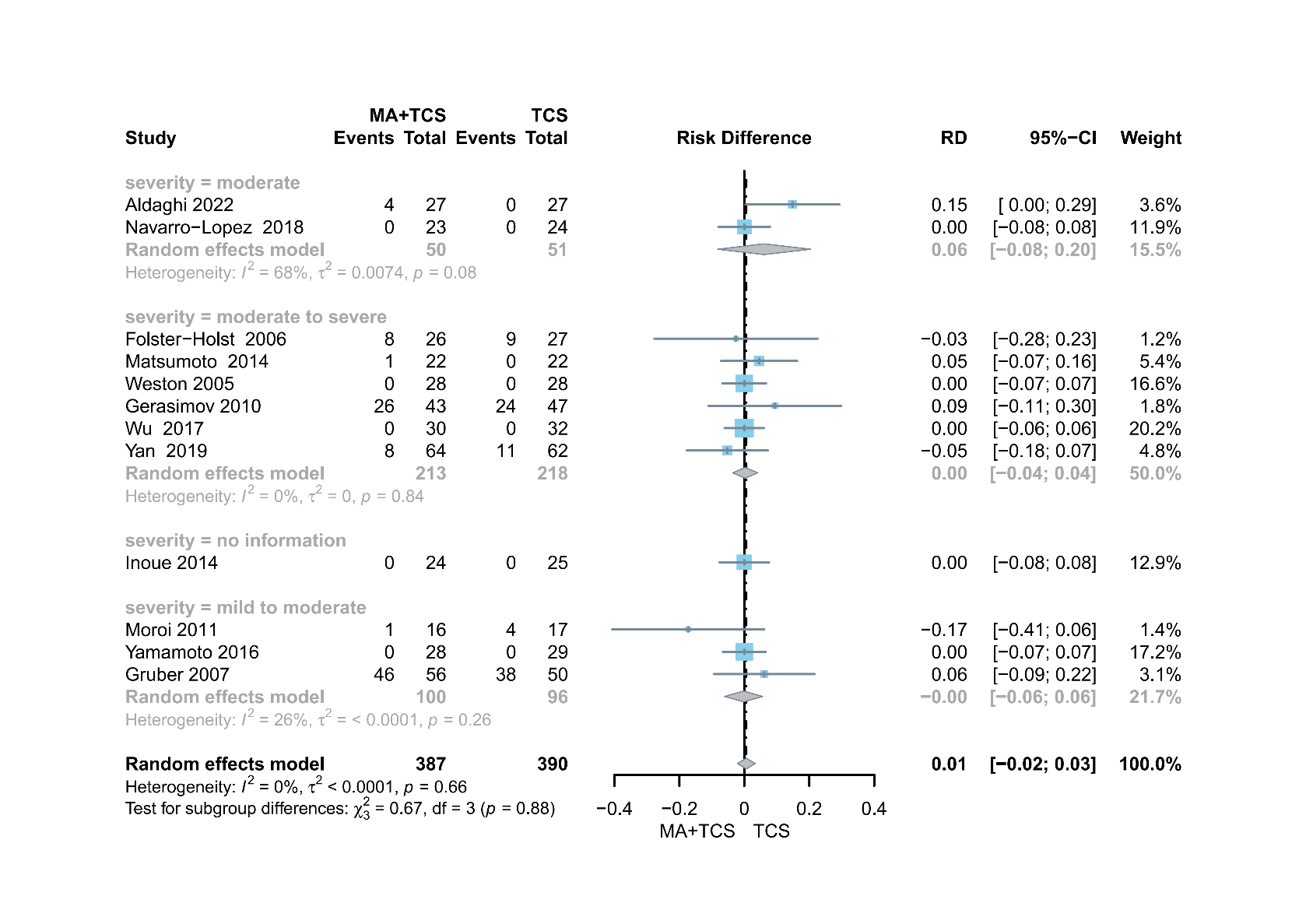


Figure S6 Forest plot of adverse events (subgroup by severities)


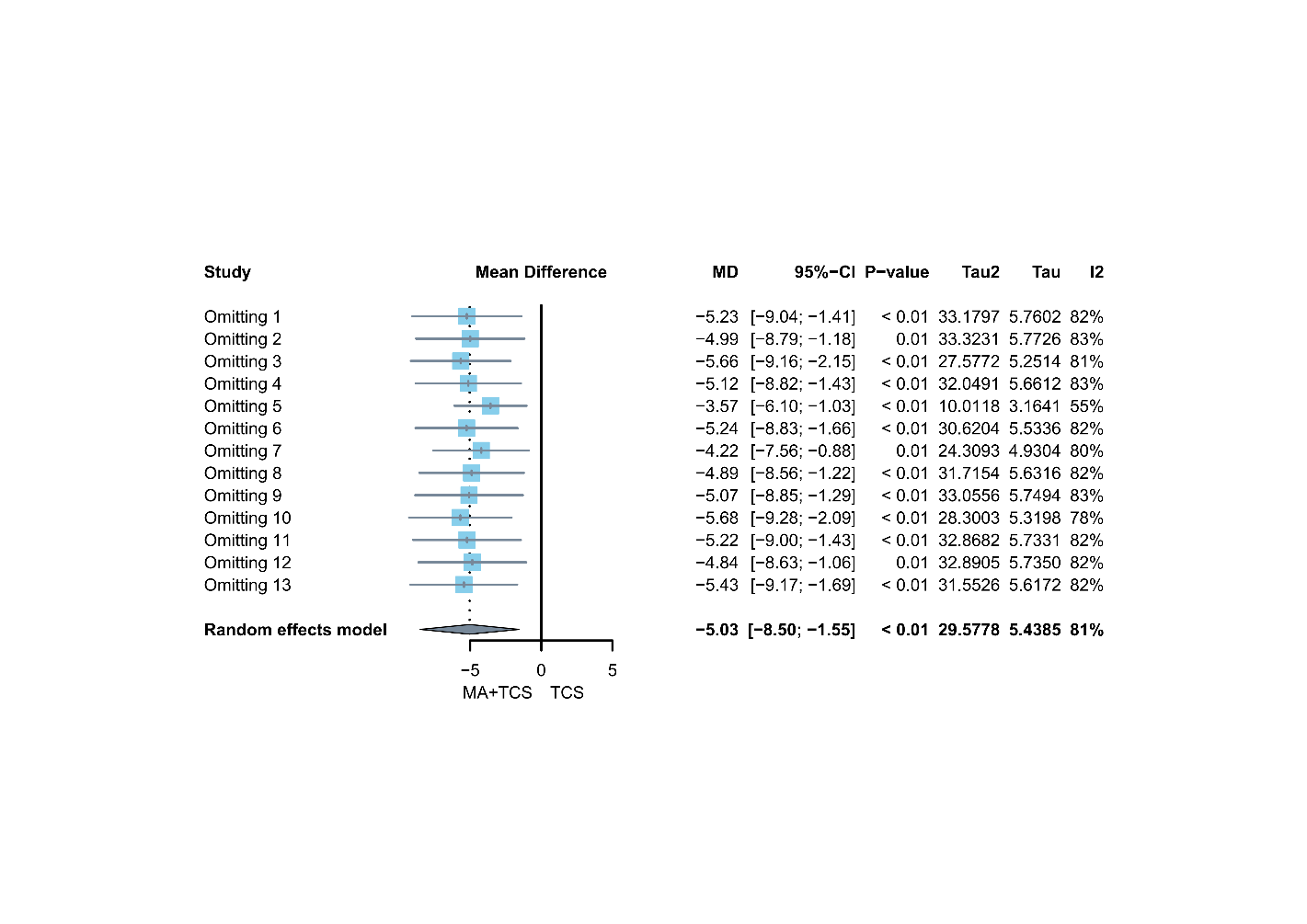


Figure S7 Sensitivity analyses of SCORSD scores
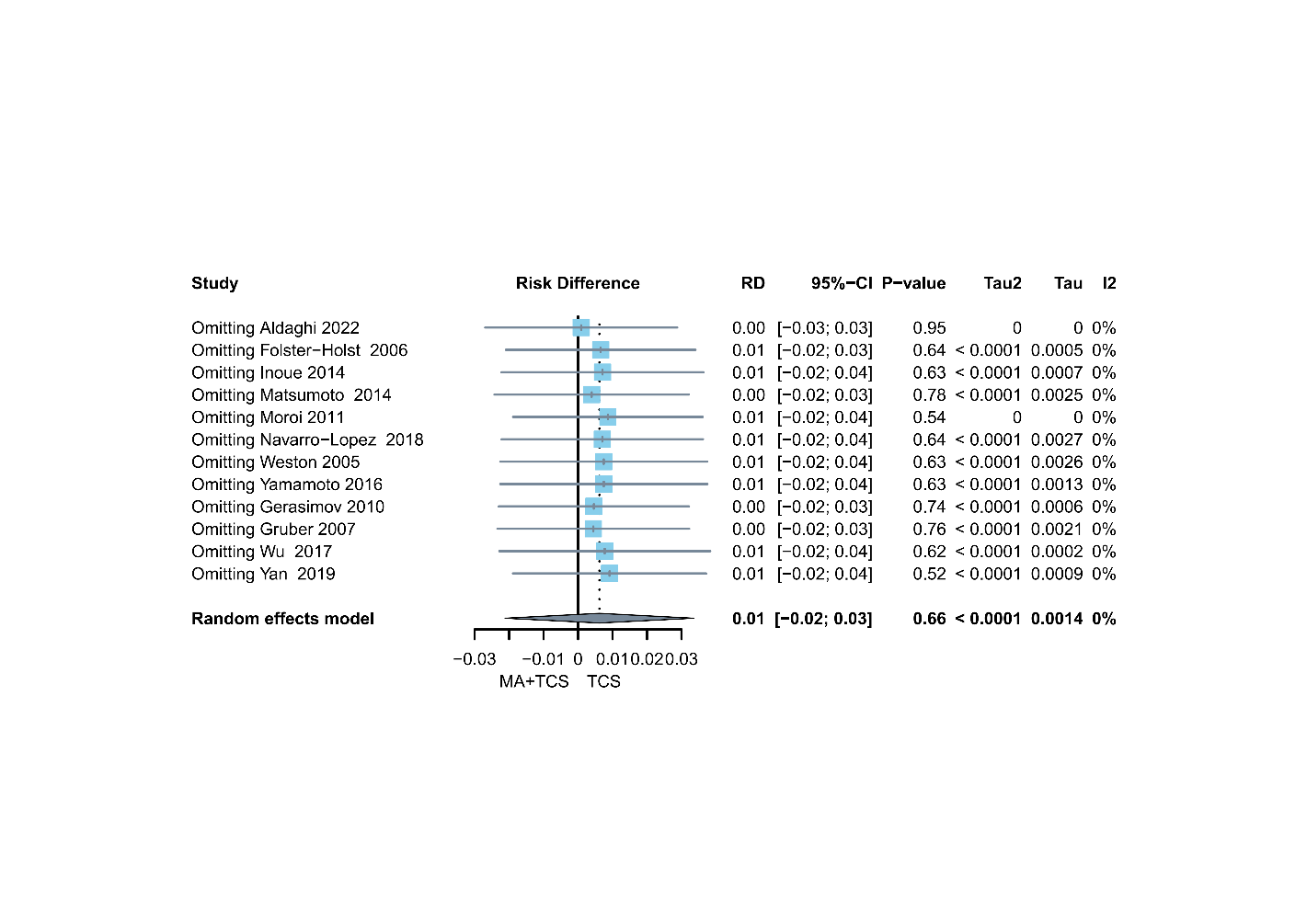


Figure S8 Sensitivity analyses of adverse events


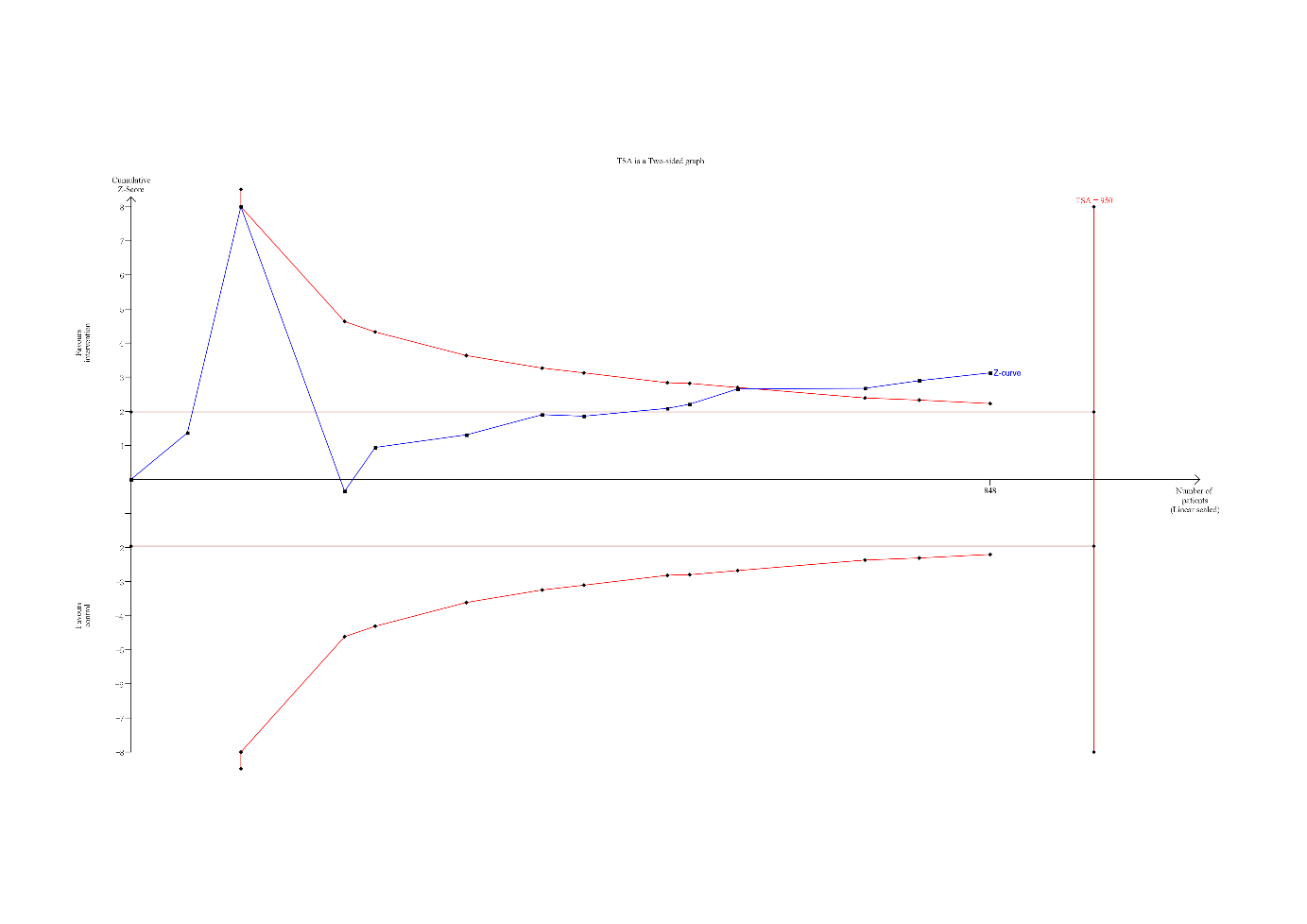


Figure S9 The plots of trial sequential analysis of SCORAD


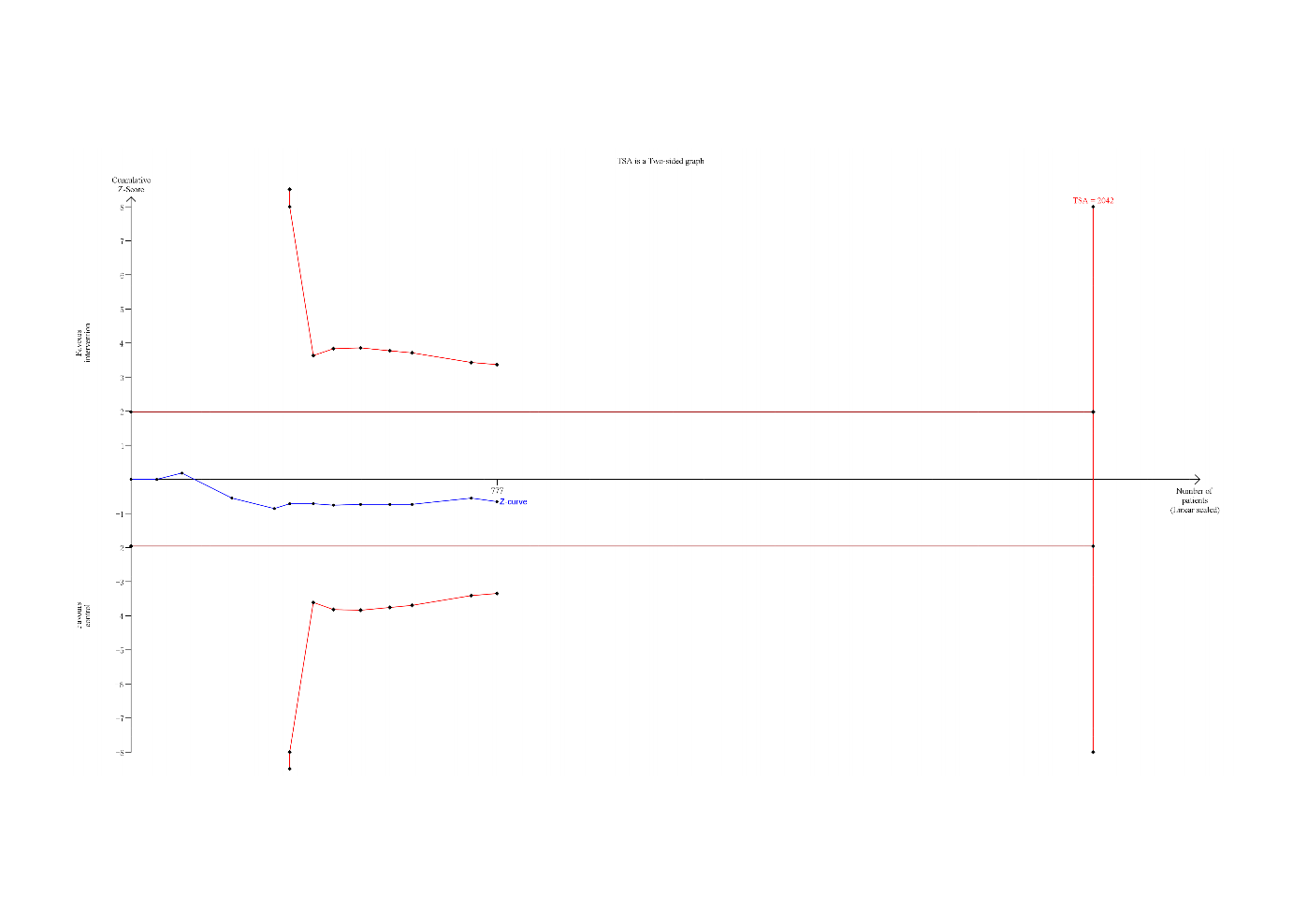


Figure S10 The plots of trial sequential analysis of adverse events


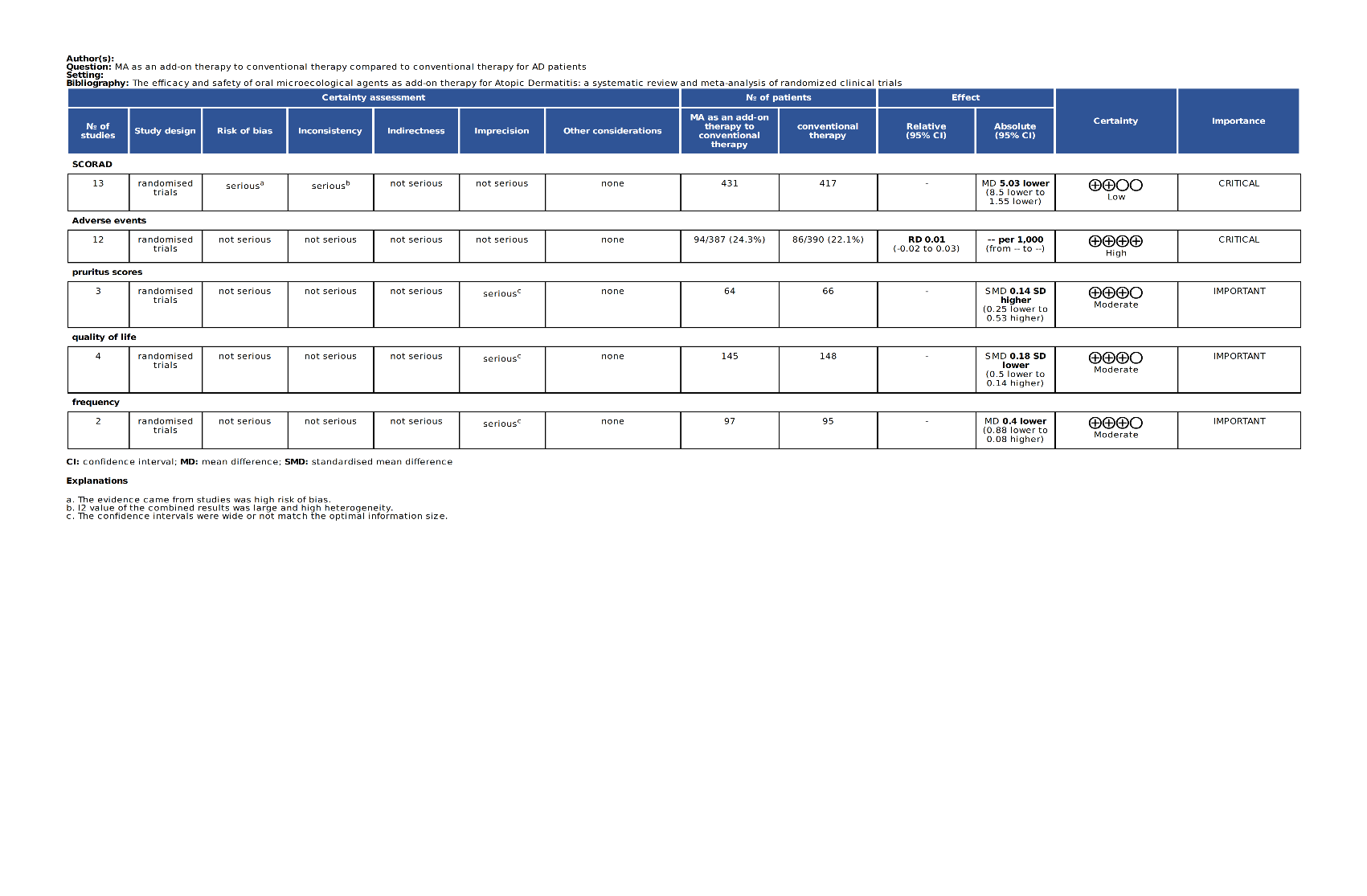


Figure S11 Results of GRADE
